# Improving quality control in the routine practice for histopathological interpretation of gastrointestinal endoscopic biopsies using artificial intelligence

**DOI:** 10.1371/journal.pone.0278542

**Published:** 2022-12-15

**Authors:** Young Sin Ko, Yoo Mi Choi, Mujin Kim, Youngjin Park, Murtaza Ashraf, Willmer Rafell Quiñones Robles, Min-Ju Kim, Jiwook Jang, Seokju Yun, Yuri Hwang, Hani Jang, Mun Yong Yi

**Affiliations:** 1 Pathology Center, Seegene Medical Foundation, Seoul, Republic of Korea; 2 Graduate School of Data Science, Department of Industrial & Systems Engineering, Korea Advanced Institute of Science and Technology, Daejeon, Republic of Korea; 3 Department of Pathology, Incheon Sejong Hospital, Incheon, Republic of Korea; 4 AI Research Team, Digital Innovation Sector, Seegene Medical Foundation, Seoul, Republic of Korea; Universiti Malaysia Pahang, MALAYSIA

## Abstract

**Background:**

Colorectal and gastric cancer are major causes of cancer-related deaths. In Korea, gastrointestinal (GI) endoscopic biopsy specimens account for a high percentage of histopathologic examinations. Lack of a sufficient pathologist workforce can cause an increase in human errors, threatening patient safety. Therefore, we developed a digital pathology total solution combining artificial intelligence (AI) classifier models and pathology laboratory information system for GI endoscopic biopsy specimens to establish a post-analytic daily fast quality control (QC) system, which was applied in clinical practice for a 3-month trial run by four pathologists.

**Methods and findings:**

Our whole slide image (WSI) classification framework comprised patch-generator, patch-level classifier, and WSI-level classifier. The classifiers were both based on DenseNet (Dense Convolutional Network). In laboratory tests, the WSI classifier achieved accuracy rates of 95.8% and 96.0% in classifying histopathological WSIs of colorectal and gastric endoscopic biopsy specimens, respectively, into three classes (Negative for dysplasia, Dysplasia, and Malignant). Classification by pathologic diagnosis and AI prediction were compared and daily reviews were conducted, focusing on discordant cases for early detection of potential human errors by the pathologists, allowing immediate correction, before the pathology report error is conveyed to the patients. During the 3-month AI-assisted daily QC trial run period, approximately 7–10 times the number of slides compared to that in the conventional monthly QC (33 months) were reviewed by pathologists; nearly 100% of GI endoscopy biopsy slides were double-checked by the AI models. Further, approximately 17–30 times the number of potential human errors were detected within an average of 1.2 days.

**Conclusions:**

The AI-assisted daily QC system that we developed and established demonstrated notable improvements in QC, in quantitative, qualitative, and time utility aspects. Ultimately, we developed an independent AI-assisted post-analytic daily fast QC system that was clinically applicable and influential, which could enhance patient safety.

## Introduction

### Background

Colorectal cancer (CRC) and gastric cancer (GC) are common types of cancer found throughout the world; they are also major causes of cancer-related deaths. According to the Global Cancer Statistics 2020, CRC ranked third in prevalence (10.0%) and second in mortality (9.4%), while GC ranked fifth in prevalence (5.6%) and fourth in mortality (7.7%) among all cancers [1]. In the United States, CRC ranked fourth in both prevalence and mortality, while GC ranked eighth in prevalence and seventh in mortality among all cancers [2]. In Korea, 243,837 new cancer cases were reported in 2018, of which, GC ranked first, with 29,279 cases (12.0%), while CRC ranked fourth, with 27,909 cases (11.4%). The crude incidence rates per 100,000 for GC and CRC were 57.1 and 54.4, respectively. For the same year, the number of cancer-related deaths was 79,153, i.e., cancer accounted for 26.5% of all-cause mortality (n = 298,777). Among all cancer-related deaths, CRC ranked third, with 8,715 cases (11.0%), and GC ranked fourth, with 7,746 cases (9.8%) [3]. Owing to the clinical significance of GC and CRC in Korea, gastrointestinal (GI) endoscopy is actively recommended as a part of the National Cancer Screening Program. Specifically, gastric endoscopy is offered as a “basic examination” every two years for adults aged ≥ 40 years, while colonoscopy is offered as an “additional examination” every year for adults aged ≥ 50 years with positive occult blood test results [4–6]. Accordingly, GI endoscopic biopsy specimens account for the highest percentage of histopathologic specimens in Korea. In particular, reference laboratories, such as our institution, are contracted for tests on specimens from primary and secondary healthcare institutions that do not have their own pathological laboratory; we handle an overwhelming number of GI endoscopic biopsy specimens, as compared to resection specimens. Moreover, GI endoscopic biopsy specimens accounted for 85.6% of all histopathologic tests performed in the past three years at our institution.

The workload of pathologists continues to increase at a steady rate; however, the number of pathologists remains relatively insufficient [7]. This relative supply shortage and increased workload among pathologists can increase human error. A diagnosis based on histopathologic test results is considered a “confirmative diagnosis” once the period of early diagnosis and treatment are missed due to a false negative (FN) result and there is no chance of recognition until the next test. Unfortunately, however, FN is the most common error in pathologic diagnoses [8]. Accordingly, there is a need for tools to help reduce FNs in a repetitive, labor-intensive, and habitual work environment or to immediately correct FNs that are detected early. A blinded review is an important and effective method for improving quality control (QC) [9]. However, it is impossible to double-check or review “all slides” that have been signed-out each day. For QC in a pathology laboratory, the general recommendation is to “randomly” review “a certain number of slides” on a monthly basis; however, this number may vary depending on the circumstances of the institution [10, 11]. At our institution, we perform blinded random reviews on approximately 12 GI endoscopic biopsy specimens per month for each pathologist as per the recommendation of the Korean Society of Pathologists [12]. Significant discrepancies may sometimes occur during this process [13] or corrections may be delayed. Nonetheless, most of these issues may be found at 1–2 months after the initial diagnosis; thus, such follow-up measures are often too late to provide any clinically significant outcomes for the patients. If artificial intelligence (AI) models could alleviate these difficulties, they would be of great benefit to both patients and pathologists.

There are several issues with implementing AI models. First, digitalization of all glass slides is required to use an AI classification model as a screening tool prior to the pathologist’s examination. The expected workflow would be as follows: glass slides are prepared; slides are digitalized (scanned); predictions are made by the AI model; positive cases are listed first, according to prediction outcomes; and pathologists open the whole slide image (WSI) via a viewer and make the primary diagnosis based on the scanned WSI, referring to the heatmap or prediction outcomes derived by AI. When necessary, glass slides would be checked directly under a microscope; in other cases, WSI alone would be used for diagnosis. Although digital pathology (DP) has undergone rapid and significant development in recent years and is quickly transitioning to clinical practice, while offering various advantages over traditional pathology [14], it cannot completely replace traditional interpretation using glass slides in pathology [15]. There are concerns regarding the detection of microorganisms, such as *Helicobacter pylori (H*. *pylori)*, in gastric biopsy tissue [15–17]. Pathology laboratories that routinely report classification and histologic grading of gastritis based on the Updated Sydney System [18] face difficulties in using DP for the primary diagnosis, without glass slides for gastric biopsy interpretation [15]. In particular, in the direct use of WSIs for the primary diagnosis, images must be scanned at a high resolution (more than 40x) to minimize discrepancies with the traditional interpretation using a microscope. In such cases, there is a significant cost associated with the establishment and operation of an information computing infrastructure for archiving and processing high-resolution WSIs. However, for QC (unlike the primary diagnosis), WSIs scanned at 20x magnification are sufficient.

Second, most pathology reference laboratories follow slide preparation immediately after interpretation; in such laboratories, changing the workflow to perform the interpretation step after scanning the slide can be difficult. Thus, some researchers have proposed the use of an augmented reality microscope (ARM) with a real-time AI integration technique instead of the WSI-based technique [19, 20]. However, regardless of the performance ability of the AI-applied ARM, there is a limit to supplementing human error by routine pathologists because lesions outside the field of view (FOV) of microscopy cannot be detected. Moreover, there are no published or commercialized ARM-based models that are applicable to GI endoscopic biopsy interpretation. Our institution applies a “rapid process” to GI endoscopic biopsy specimens, in which pathologists perform readings as soon as slide preparation is completed. Therefore, the application of a WSI-based AI model for screening at our institution is not suitable, as it is not a rapid reporting system.

Lastly, using an AI model to recommend the priority for interpretation, provide visualization of suspected lesions, and expect classification outcomes prior to interpretation, could induce the risk of an AI-dependent bias by pathologists. Bias is problematic, and it can also induce dependency owing to the superior performance of the AI model. This would certainly be a concerning issue from the patient’s perspective. In case of a misdiagnosis due to bias by a pathologist who is dependent on a high-performance AI model (e.g., if both the AI model and pathologist overlook a lesion or if the human pathologist overly relies on a negative prediction by the AI model and signed out the slides without checking the WSIs), a dispute may occur regarding legal responsibilities [21]. Numerous researchers have developed and proposed various histopathologic WSI-based AI models. Moreover, some studies have claimed that their models demonstrate performance comparable to, or better than that of, human pathologists [22–25]. However, pathologists bear all legal responsibilities and authority for the diagnostic outcome in each case, and AI cannot completely replace pathologists [26]. In particular, the differentiation of carcinoma/high grade dysplasia (HGD) in GI pathology is prone to inter-observer variability or discrepancy depending on the group [27, 28]. Accordingly, it is easy to predict variability or discrepancy in the performance of AI classification models based on how the classification was defined in GI pathology. Other similar studies have also shown differences in the definition of classification according to group, while some excluded gray-zone diagnoses from their studies [29–31]. Furthermore, in many cases, pathologists use ambiguous expressions in GI endoscopic biopsy (or other small biopsy) specimen reports in their daily practice, unlike in the interpretation of resection specimens, such as, “atypical glandular proliferation of undetermined significance (AUS),” “suspicious for dysplasia,” “malignancy cannot be ruled out,” and “favor neoplastic.” AI models cannot possibly resolve these pathology ambiguities; there are inherent limitations to small biopsy interpretation [32].

Many studies have examined the accuracy with which AI models can diagnose a region of interest, focusing on improving their performance. However, this can sometimes cause low reproducibility in clinical implementations, as the conditions for high accuracy in AI models are significantly different from those in daily real-world practice [33, 34]. Furthermore, highly refined data preparation is important for ensuring high accuracy in AI models. Poor scan quality (out of focus, tissue missing, or air bubbles) and poor slide quality (poor staining, poor sectioning, tissue artifacts, air bubbles, tissue folding, or poor dehydration) lower the performance of models [35]. Accordingly, their performances can be optimized through training using highly refined data, with all artifacts artificially removed, and by scanning well-prepared slides, with no blurs. However, this may be contradictory and unrealistic considering the transition to a fully digitalized pathology laboratory. As mentioned above, the number of histopathologic tests is gradually increasing [7], increasing the workload of pathologists and overloading histopathology technicians who prepare the slides [36]. Increased workload could affect the work competency of histopathology technicians and degrade the slide quality [37, 38]. Thus, pathologists cannot control the quality of all slides, and in daily real-world practice, they encounter a much higher percentage of slides that do not meet the stipulated quality standards. The same conditions apply to scanning quality. Scanner companies declare a scan error rate of 1–3%. At our institution, the average percentage of slides that are rescanned after being processed as an “error” is 2.4%. These slides are of poor quality, rendering them difficult to interpret; WSIs that are somewhat interpretable, but are of poor quality with various artifacts mentioned above are found much more commonly and frequently. Rescanning all “interpretable but poor quality” WSIs is impossible and expensive. Thus, in the development and application of usable AI models, it would be more appropriate to develop and apply “reliable” AI models that reflect reality for routine practice, rather than dealing with an inefficient work burden simply to maximize the AI model performance.

### Objectives

Our goal was to develop AI models with reliable performance that can reduce potential human errors (especially FNs) by pathologists–but not necessarily be better at diagnosis than pathologists–without infringing on or threatening the diagnostic responsibilities of pathologists and maintaining the standard microscopy workflow. We aimed to apply such models as a tool for daily fast QC, as the most realistic and suitable method in routine practice for GI endoscopic biopsy interpretation. Our primary rules for the development of AI models were as follows: (1) FNs caused by potential human errors in daily practice are detected and enable immediate actions to prevent the neglection of patients who require treatment and promote patient safety enhancement; (2) artifacts and variability in the field are reflected, promoting applicability to the daily real-world practice; and (3) the unique authority of each pathologist is respected, as a reference for accurately differentiating between “neoplastic and regenerative” and between “cancer and dysplasia” is not our purpose. While many high-performance AI algorithms have been previously developed, there are very few cases of such algorithms being used in daily practice during pathologic diagnosis [26]. Therefore, we aimed to develop AI classification models, and clinically implement them, for daily QC in the histopathological interpretation of GI endoscopic biopsy.

## Materials and methods

### Development of AI models

#### Data preparation

This study used GI endoscopic biopsy specimens with confirmed diagnoses that were stored at Seegene Medical Foundation (SMF), Seoul (Seongdong -gu, Seoul). These specimens were selected from specimens that SMF had received from approximately 400 local clinics and hospitals throughout Korea for histopathologic diagnosis. To use these specimens for research purpose, we obtained approvals from the Institutional Review Board (IRB) of SMF (IRB Approval number: SMF-IRB-2020–007) and IRB of Korea Advanced Institute of Science and Technology (KAIST) (IRB Approval number: KAIST-IRB-20–379). All experiments were conducted in accordance with the relevant guidelines and regulations given by the two IRBs. All patient records were completely anonymized, and all data were stored and analyzed only in SMF servers. This study corresponds to the guidelines for performing laboratory quality control and test method evaluations, such as accuracy tests, using remaining human-derived specimens after treatment and diagnosis and received waiver of informed consent. Among the specimens stored at SMF after diagnosis between June 2018 and April 2021, 3,979 gastric biopsy and 1,848 colorectal biopsy hematoxylin-eosin stained slides were randomly selected and scanned (Panoramic 250 Flash III, 3DHISTECH, 20x). Of these, some were excluded for various reasons during the study (to be described later); ultimately, 1,762 gastric biopsy and 1,509 colorectal biopsy WSIs were used to develop a classifier model. The data were divided into training (80%), validation (10%), and test (10%) based on the WSIs. For accurate training and evaluation of the model, the training, validation, and test data were divided at patient level (i.e., whole slide) with no overlap. Table 1 shows in detail how the WSIs were specifically divided into training, validation, and test data. To develop AI classification models for daily QC in real-world practice, we used raw data, preserving routine artifacts and interobserver variability, to strictly reflect daily practice, rather than artificially establishing well refined WSI data sets for high model accuracy.

**Table 1:** Details of the datasets used for the development of each AI model.

Cases (WSIs)	Gastric biopsies				Colorectal biopsies			
	Classes			Total	Classes			Total
	M	D	N		M	D	N	
**Training**	284	472	613	**1369**	209	432	568	**1209**
**Validation**	70	70	103	**243**	50	50	50	**150**
**Test**	50	50	50	**150**	50	50	50	**150**
**Total**	404	592	766	**1762**	309	532	668	**1509**

**Abbreviations: AI** (artificial intelligence), **WSI** (whole slide image), **M** (Malignant), **D** (Dysplasia), **N** (Negative for dysplasia)

#### Ensuring diagnostic reliability with usual interobserver variability

In total, five pathologists with at least five years of clinical experience and specialization in pathology (MJK, YRC, SHL, YMC, and YSK) made annotations, and all WSIs were first reviewed by each annotator. If an annotator disagreed with an existing diagnosis, at least two pathologists reviewed the applicable slides together. If at least two pathologists disagreed with the existing diagnosis, the slides were excluded from the data collection set. Thus, cases of disagreement by only one pathologist were considered to reflect realistically acceptable interobserver variability, and annotations made by those who agreed with the existing diagnosis were included as training data. The annotation rules are provided in the S1 Methods.

#### Reflecting the qualitative environment of pathologic interpretation in the real world

The quality of the slides was not adjusted for optimal model accuracy. In fact, routine artifacts (e.g., poor sectioning, poor staining, poor fixation, and air bubbles) were used in training because they lacked artificial improvement (re-preparation high-quality slides just for training). However, poor-quality slides that were difficult for pathologists to interpret were excluded.

Scan quality was also not adjusted artificially. Scanner manufacturers declare an average error rate of 1–3% for slides that require rescanning, while others are mostly minor problems that do not affect the interpretation; these include some small out-of-focus areas, overlapping tiles, and some scanned tissues with cropped edges. To reflect these routine minor errors, artificial improvement measures, such as rescanning to obtain high-quality WSIs, were not implemented for training only. Moreover, poor-quality WSIs that would be difficult for pathologists to interpret were excluded.

#### Definition of each class for training and internal validation

Model development was attempted for daily QC in all GI endoscopic biopsy specimens at our institution; thus, initially we used four categories (malignant [M], dysplasia [D], negative for dysplasia [N], and uncategorized [U]) to classify all possible diagnoses. Subsequently, we started the development of a quaternary classifier model based on these classes (Table 2). However, the inclusion of class “U,” which by definition had the highest heterogeneity among diseases, degraded the performance of the model [39] and was determined to be ineffective in daily practice. Accordingly, a ternary classifier model that excluded class U was selected for the trial run; thus, cases corresponding to class U were excluded from the training dataset for development of the ternary classifier model.

**Table 2:** Definition of each class for training and internal validation.

Class	Definition	Color
**M** *(Malignant)*	Diagnosed as malignant neoplasm, including adenocarcinoma, suspicious for (s/f) adenocarcinoma, suggestive of (s/o) adenocarcinoma, (s/f, s/o) high-grade lymphoma, and any other (s/f, s/o) carcinoma or malignant neoplasm.	Red
**D** *(Dysplasia)*	Diagnosed as dysplasia, including (s/f, s/o, favor) adenoma with dysplasia of **any grade**.	Blue
**N** *(Negative for dysplasia)*	Diagnosed as non-neoplastic mucosal lesion, including inflammation, ulcer, and polyps.	-
**U** *(Uncategorized)* Finally excluded	The remaining lesions that do not fall under the aforementioned three classifications; for example, atypical glandular proliferation of undetermined significance, indefinite for dysplasia, **(s/f, s/o) neuroendocrine tumors (grade 1 or 2)**, submucosal tumors, (s/f, s/o) low-grade lymphoma, and (s/f, s/o) stromal tumors.	Green

#### Training of AI models

**Fig 1:**
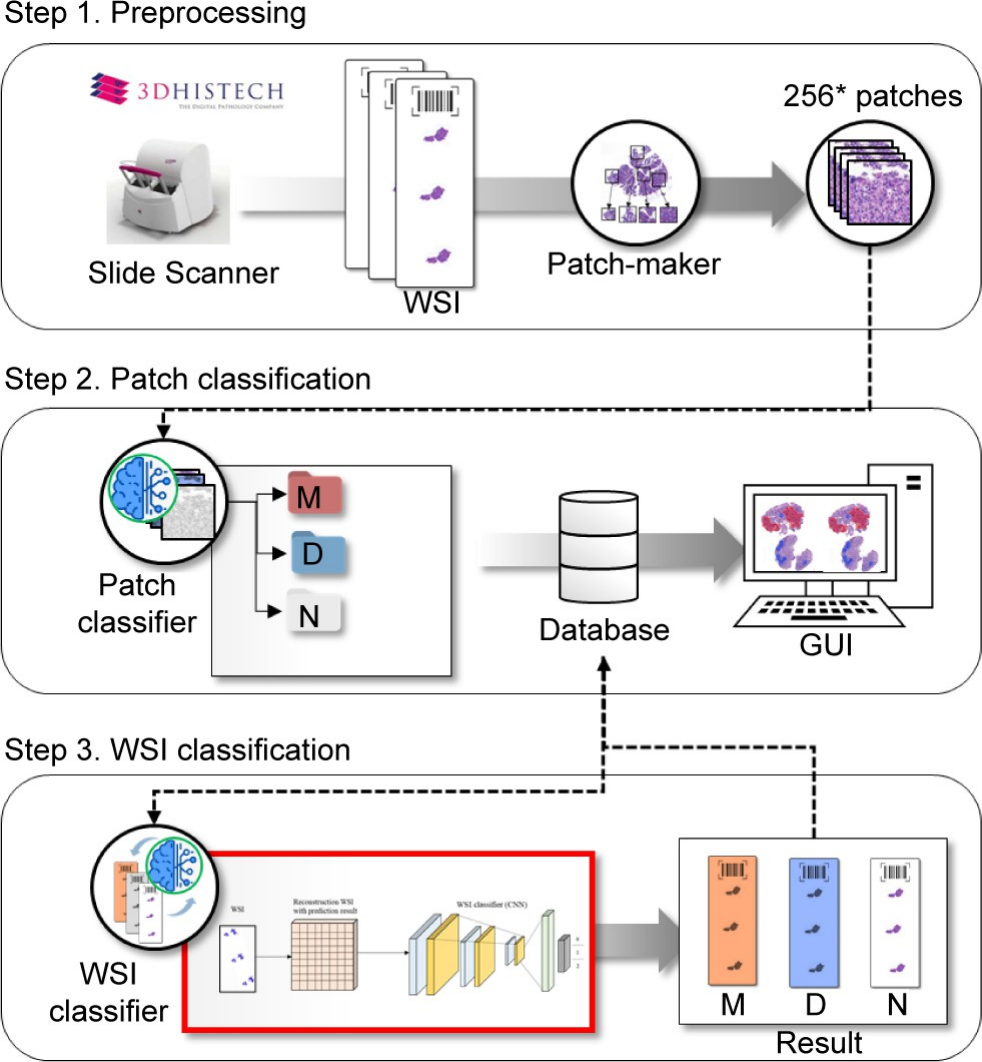
Overview of the training of the AI model for daily QC. An overview of the modeling method used, which includes three steps, is shown. The first step is the data preprocessing step; data are organized and converted in several patches that can be used to train the models. The next step is the patch classifier training step; training is carried out based on the labeled data for each patch generated from a single WSI. The final step is the training WSI classifier step; models that generate information for classification of WSIs are trained by combining the data regarding each WSI. The same model training method was applied to gastric and colorectal models. Abbreviations: AI (artificial intelligence), WSI (whole slide image), QC (quality control), GUI (graphical user interface), M (Malignant), D (Dysplasia), N (Negative for dysplasia).

WSIs include giga pixel-level data; thus, they are not suitable to be used all at once for deep learning (DL). Accordingly, WSIs that include giga pixel-level data must be suitably converted to be processed by the models. Model construction was based on a method of converting and processing WSI into patches, similar to that used in various existing studies [40, 41]. As mentioned in numerous studies on convolution neural network (CNN)-based WSI processing [42, 43], the complexity of the CNN can cause a loss of data and resources from WSIs when they are processed all at once. Consequently, we conducted model training in three steps: the data preprocessing step, patch classifier training step, and WSI classifier training step (Fig 1). The same model training method was applied to gastric and colorectal models.

#### Data preprocessing

The first step in AI model training was the data preprocessing step. Model training was conducted based on ground truth data generated by the method mentioned in the previous section and labeled data as shown in Table 1. Data were set up without annotation for class N WSIs and with annotations for class M and D WSIs. The WSIs were saved in mrsx format, and data preprocessing was conducted with Openslide’s library [44].

The method used in our study largely required two deep neural network (DNN) models for WSI learning. To train each model, data were prepared in two forms: WSI data and patch data. For WSI data, the scan configuration values of the collected images were checked and images with different values were excluded from the dataset. This was to prevent images scanned under different conditions from causing errors in the model. The WSI data set finally selected is shown in Table 1. The next step was patch data preprocessing. For class N, Openslide’s library was used to convert a whole slide to multiple patches, each of which contained a 256x256 pixel image (image with a width of 256 pixels and a height of 256 pixels) derived from the WSI. The patch size was determined in consideration of the trade-off between user convenience and performance. More specifically, using the same set of slides, we generated alternative patches of 128×128, 256×256, 512×512, and 1024×1024 pixel images, which are typical input image sizes for CNN, and then compared the actual classification performances, while seeking user feedback on the size appropriateness for the user interface (UI). For example, 1024×1024 and 512×512 size pixel images sometimes produced slighty better performance but were considered unsuitable for determining the lesion location information provided on the UI (refer to “Development of SeeDP”). In addition, with the 128x128pixel images, the classification model performance was noticeably deteriorated. Thus, the 256×256 pixel image patch size was considered adequate. For classes M and D, only patches within the annotations were selected and saved; patches outside the annotations were not included in the dataset.

**Fig 2:**
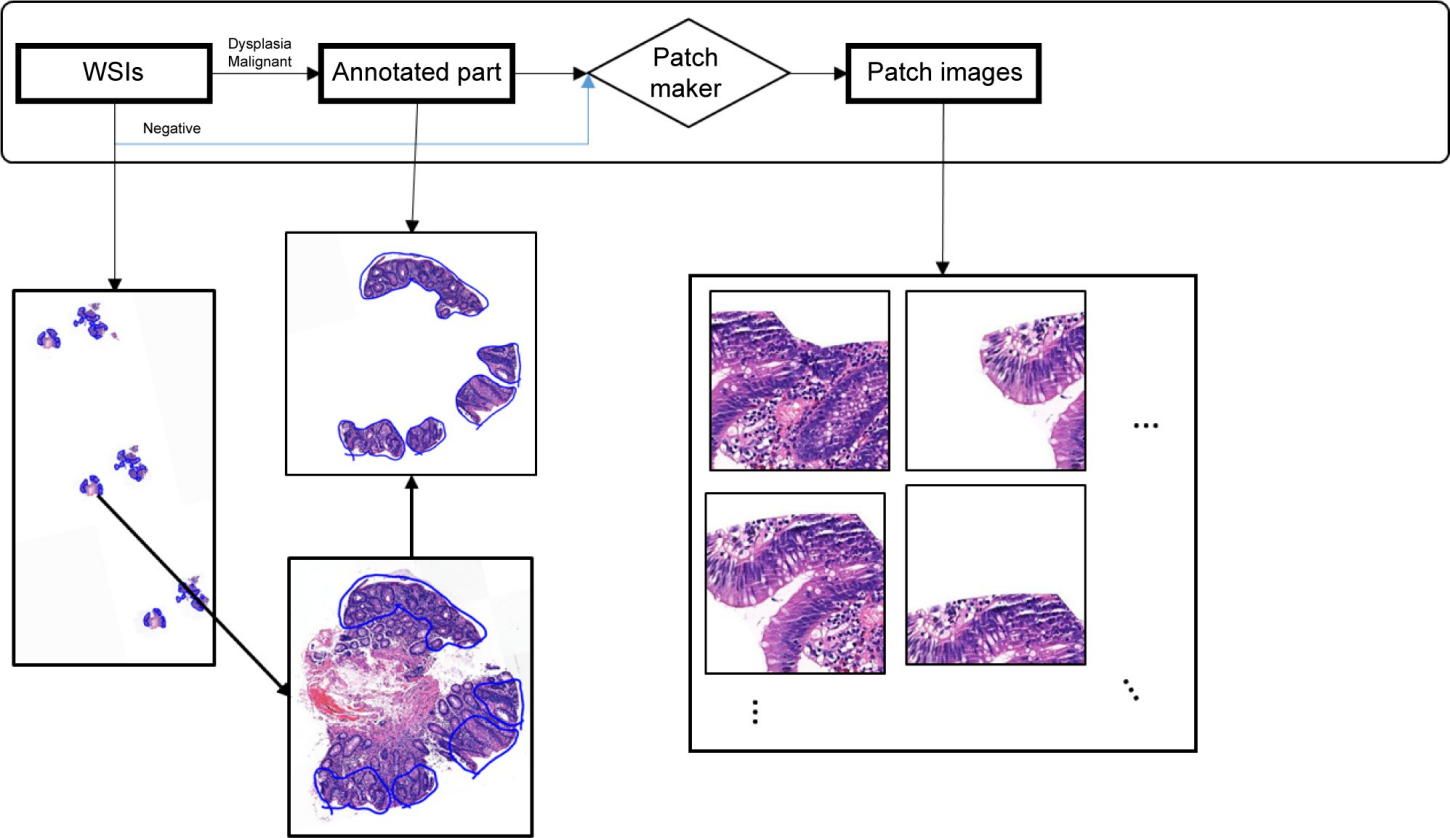
Data preprocessing workflow. The workflow for generating patch images during the data preprocessing step is shown. This workflow involves a series of processes for generating patch images based on the annotation data with WSIs loaded into Openslide’s library, which were processed differently according to class. Abbreviations: WSI (whole slide image).

For an accurate assessment of the model performance and feedback, training, validation, and test datasets were constructed based on the WSIs and their associated patches. If the training, validation, and test datasets were constructed without considering the association between WSIs and patches, patch images generated from the same WSI could be found simultaneously in the training dataset and test or validation dataset. Lastly, patch data were randomly sampled from the generated training pool to balance and minimize bias in the data classifiers (Fig 2).

#### Model training (patch classifier)

**Fig 3:**
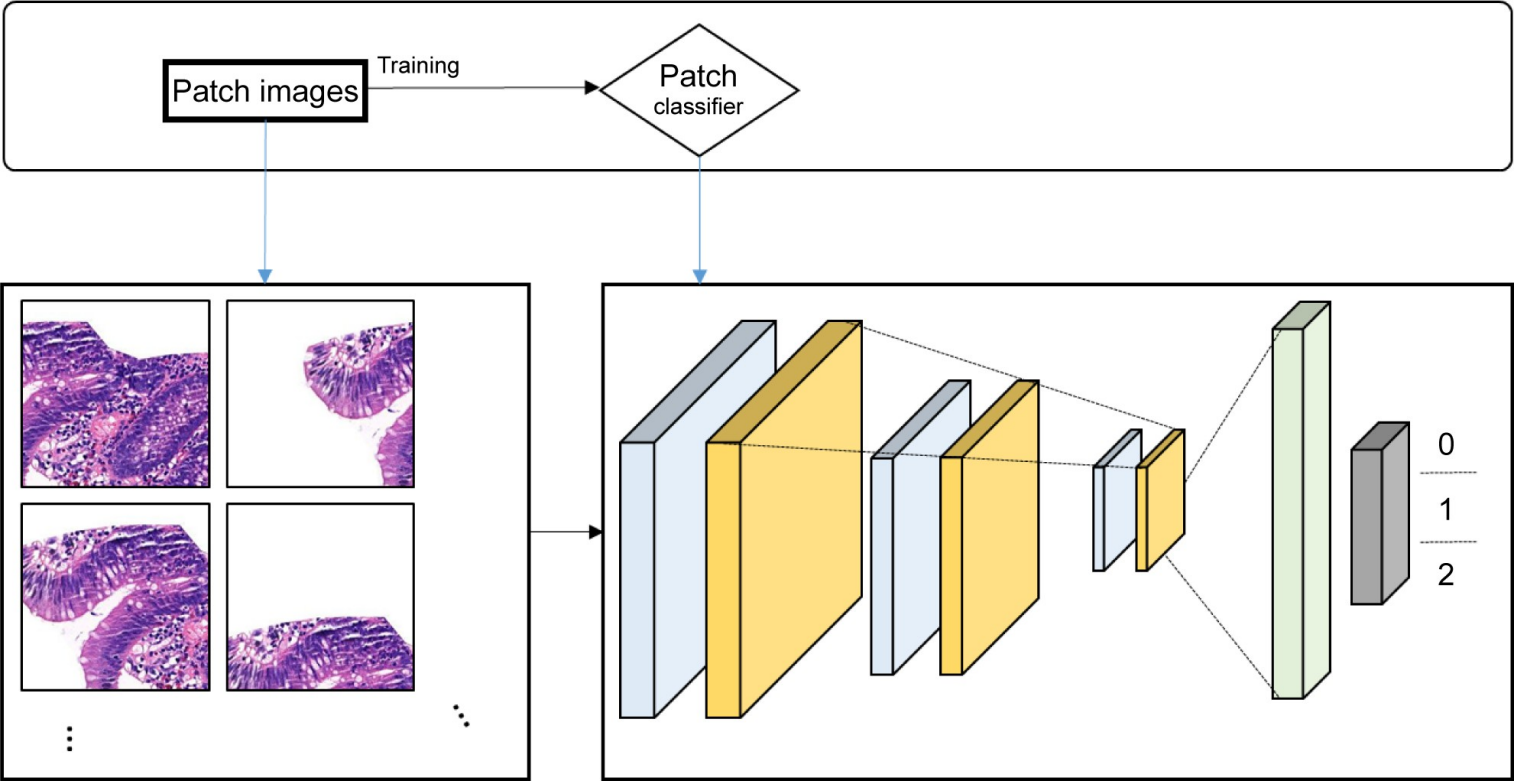
Concept of patch classifier training. The conceptual diagram of the patch image training process is shown.

In the second step of AI model training, i.e., the patch classifier training step, CNN-based DNN architecture was used to train the image classifiers. CNN has shown remarkable achievements since the development of AlexNet [45]. In particular, combining DL based on CNNs has led to its active use in the medical domain for fields dependent on human intervention, such as segmentation and detection [46–49]. For patch image classification, we used DenseNet201, which showed excellent performance in DNN architecture for model [50]. DenseNet is well recognized for its remarkable performance in competitive object recognition benchmark tasks, such as ImageNet and CIFAR-100 [51, 52]. DenseNet inputs the connect feature-maps generated in all previous layers to subsequent layers and achieves easy training and parametric efficiency by reusing the features. Using this method, deep layers of the network can reuse the features by accessing all connected feature-maps generated in previous layers [53]. We inputted training data that were setup in the pre-trained DenseNet201 model. Patch classifier model was trained to accurately infer three correct labels (classes M, D, and N) for each patch image (Fig 3). Moreover, in order to improve the effectiveness of the training process, noise data were removed based on the training loss value of the DenseNet201 model [39].

The classification results generated from the patch classifiers had two purposes: 1) to be used as elements for inference by the WSI classifiers and 2) to provide explanation in the UI. In particular, WSIs were trained, based on the model trained in this step, for use as elements for WSI classifiers. Patch classifiers can generate classification results by distribution in each class, which can be used as core information.

#### Model training (WSI classifier)

WSI classifiers were trained with the patch classification results, which were fragmented data of WSIs, as elements for inference by correct WSI labels. In the WSI classifier training step, WSI classifiers were trained through a series of processes based on 1) the patch maker model used in the data preprocessing step, and 2) the patch classifiers from the model training step. This allowed for the efficient use of resources needed for WSI classification, which included giga pixel data, and each model was designed to operate dynamically like a single model.

**Fig 4:**
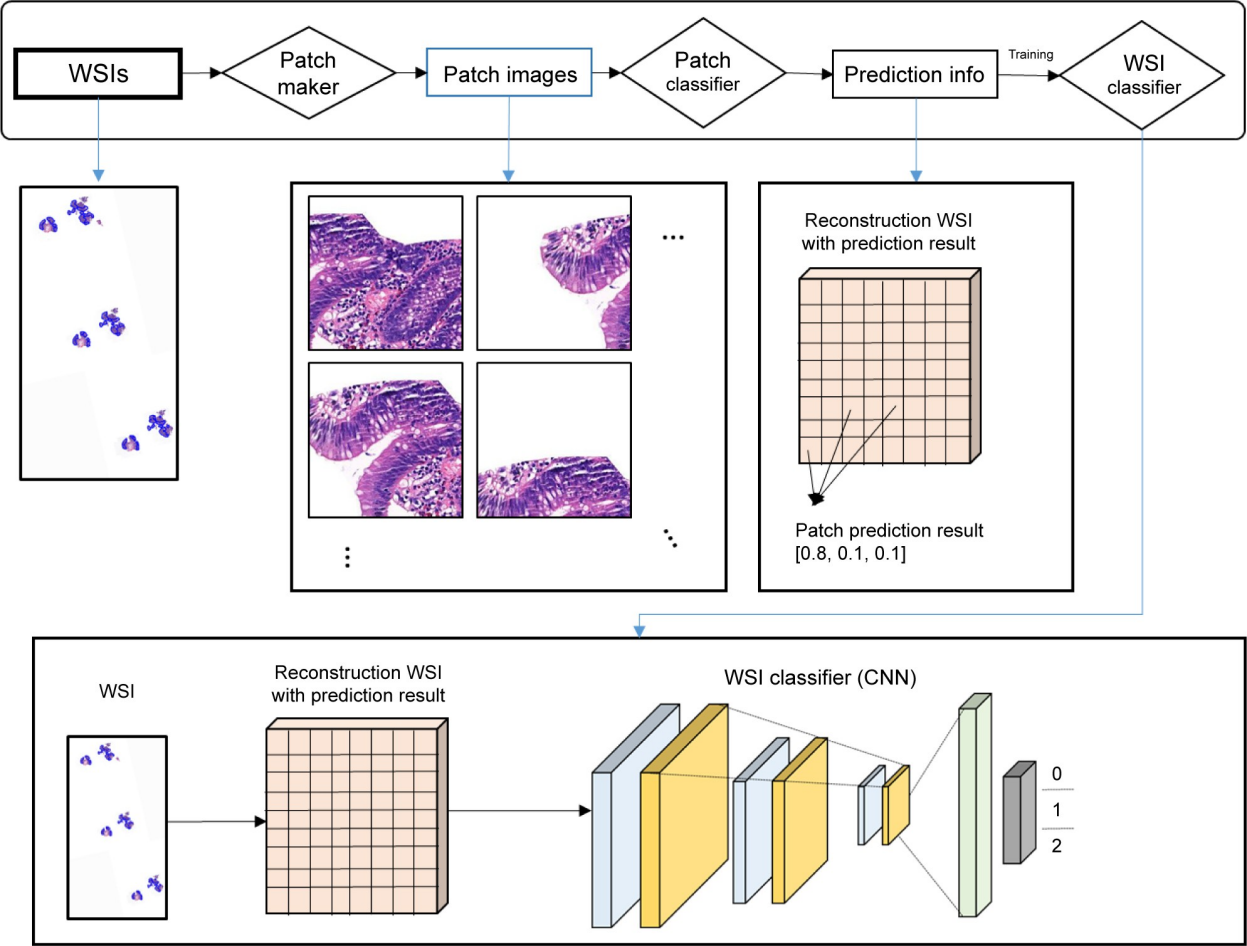
Training process for WSI classifiers. A conceptual diagram of the training process for WSI classifiers is shown. A single WSI is converted into several patch images by the patch maker and distribution information is generated by the patch classifier, which is then converted to a reconstructed WSI. Reconstructed WSIs typically have images rectangular in shape. To classify the reconstructed WSIs, we used a trained CNN model as the WSI classifier. This series of processes was applied to both the gastric and colorectal models to generate two models. Abbreviations: WSI (whole slide image), CNN (convolution neural network).

In the WSI classifier training process (Fig 4), WSIs were converted to patch images by the patch maker, and converted patch images were reconstructed based on the locational information to train WSI classifiers. This WSI classifier training process largely included three steps: the patch maker step, patch classifier step, and WSI training step. In the patch maker step, WSI were converted to several patch images. To preserve the location information of each patch image during this conversion process, index and location information of the converted patch and the WSI from which the patch was generated were recorded in a database (DB). In the patch classifier step, classification information of each patch from a single WSI was inferred through the trained patch classifier. The inference information of each patch image was stored in the DB as per the index of the patch image. Accordingly, the index, location, and classification information of each patch image of a single WSI were stored in the DB. Ultimately, the DB of patch images was combined for WSI training, and reconstructed WSIs were generated. Reconstructed WSIs included a condensed version of the characteristic information of the WSI; thus, this information can be processed in CNNs, in a similar manner as that for a single image. We trained the slide classifier to classify the reconstructed WSIs using the same architecture as the patch classifier DenseNet201. In other words, the entire process followed a process that utilized the DenseNet architecture to produce patch classification results to generate a distribution of predicted confidence values for each patch image and to train another DenseNet to classify the reconstructed WSIs. This series of processes was applied to both gastric and colorectal models to generate two models.

### Operationalization of AI models

**Fig 5:**
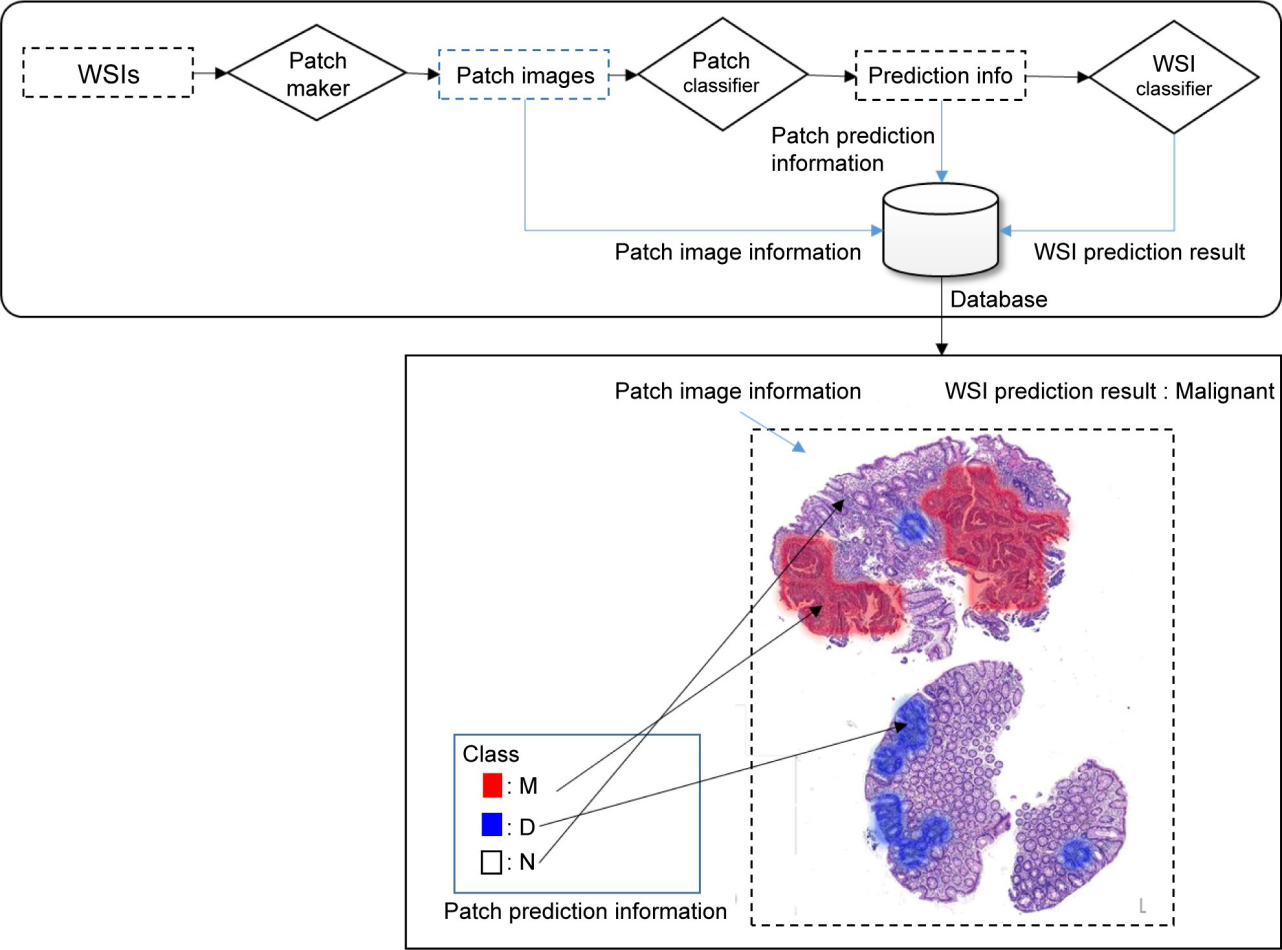
Workflow of the operationalization of our trained models. The operationalization of the trained WSI classifier model is similar to the training workflow. When a new single WSI is loaded into the trained WSI classifier model, the patch maker generates multiple patch images from the WSI, along with patch information, such as the index and location information, which are then recorded in the database. Each patch image is inputted into the patch classifier and the class is inferred by the model. Thus, the classification result is generated and stored in the database. Ultimately, the WSI classifier combines the patch image and database information to generate the reconstructed WSI and classification. This process includes the integration of classification information and location information of each patch for conversion to the reconstructed WSI, as well as input into the WSI classifier for generating WSI classification results and database storage. Therefore, the database stores three types of information: the patch image, patch-level classification, and WSI-label classification information. Patch image information includes the patch index, patch image, and patch location information; patch-level classification information refers to the class inference results generated from the patch classifier model; WSI level classification information refers to the class information inferred by the WSI classifier model for a single WSI. Abbreviations: WSI (whole slide image), M (Malignant), D (Dysplasia), N (Negative for dysplasia).

In this step, the entire prediction framework was operationalized, based on the model training in previous steps, to generate results and additional information. The prediction method was similar to the training order, with the patch maker followed by the patch classifier and WSI classifier. The models in our study were developed and trained separately for gastric and colorectal biopsy specimens. Each model operated in the same order and supported the DP solution we developed. Fig 5 shows the workflow of the trained models in operation.

### Development of SeeDP (Seegene medical foundation’s AI-assisted DP total solution)

We needed an integrated solution to support a series of processes, from the scanning of daily signed-out glass slides for conversion to WSIs to automatic-AI prediction, visualization of results, and information processing. Accordingly, we developed “SeeDP” (AI-assisted DP Total Solution), which is a comprehensive software that can serve as a WSI viewer and also perform AI prediction visualization, and analyze and process the relationship between the pathologic diagnosis and AI prediction. We developed SeeDP as an open source-based WSI viewer that enables pathologists to use a web browser to check WSIs that have undergone AI prediction. This viewer was produced using Openslide’s library and OpenSeadragon3.0.0 (GitHub, San Francisco, California, United States) [44]. We enabled the qualitative assessment of the results of the AI model (heatmap and prediction) recorded in each WSI list. These assessment data can be used as additional data to strengthen the performance of future models. Additionally, we established scanner operating guidelines, daily QC procedures, and response measures for system errors to build a daily QC system suitable for daily practice.

Details of SeeDP are presented as Supporting Information (major equipment and specifications are presented in S1 Table, and the main features are presented in S1–S6 Figs).

### Workflow of the SeeDP daily QC system

**Fig 6:**
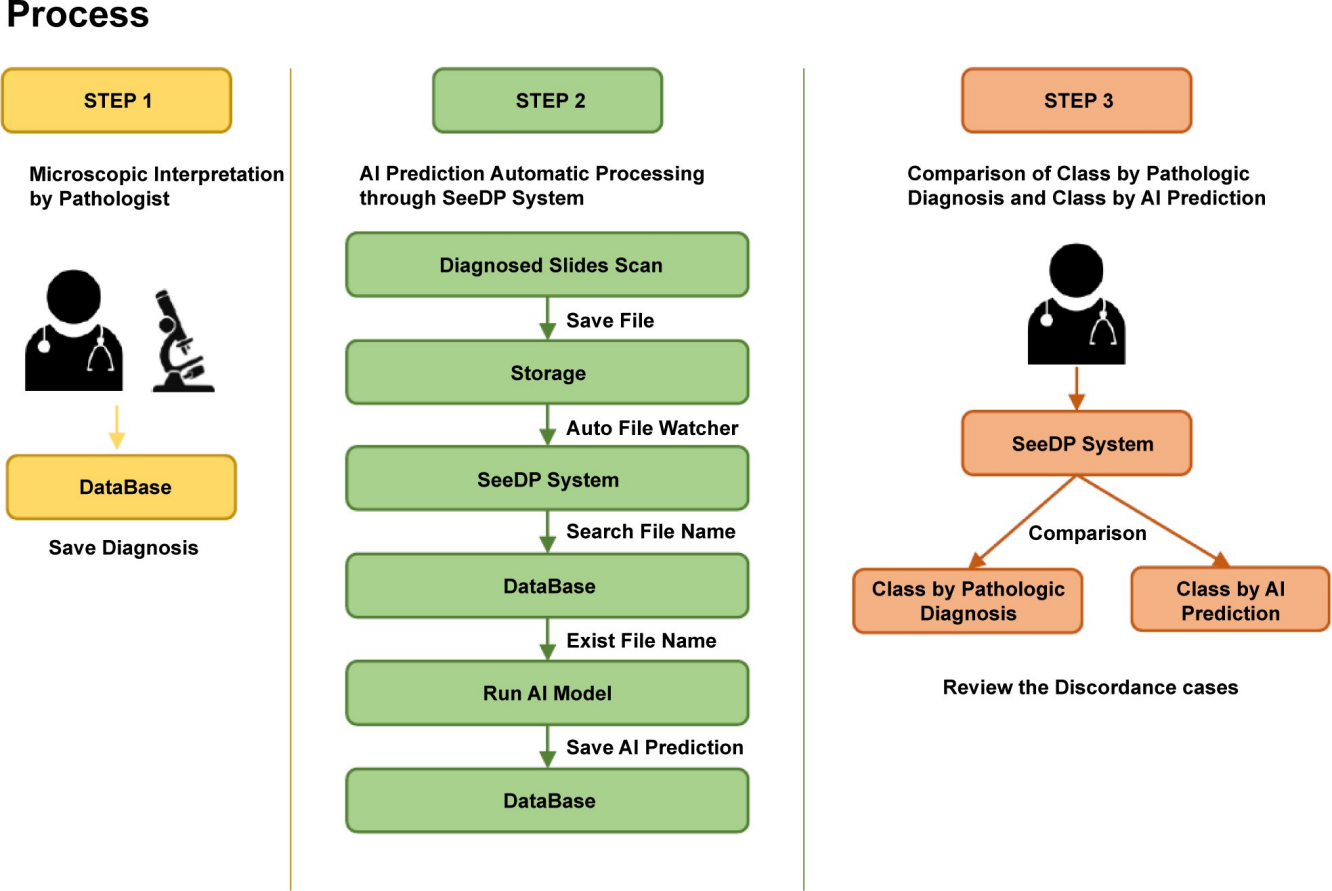
Workflow of the SeeDP daily QC system. In routine practice of gastrointestinal endoscopic biopsy reading, the pathologist first performs the microscopic interpretation. Next, the signed out slides are scanned, then in SeeDP system, their file name (pathology number) is searched for in the database, and the AI model corresponding to each organ is determined and implemented. Finally, via the SeeDP program, scanned slides are organized into classes by pathologic diagnosis and by AI prediction. Pathologists can access the SeeDP system to check whether there is concordance between each class and review discordant cases preferentially. Abbreviations: AI (artificial intelligence), QC (quality control), GUI (graphical user interface), SeeDP (Seegene Medical Foundation’s AI- assisted Digital Pathology Total Solution).

The AI-assisted daily QC system can perform daily scans and AI predictions on all GI endoscopic biopsy slides that have been signed out after microscopic interpretation. The following system workflow was designed and applied for optimal use in real-world practice (Fig 6). In step 1, the pathologist performs the microscopic interpretation. In routine practice of GI endoscopic biopsy reading, the slides are not prepared separately for different types of specimens (e.g., gastric biopsy, colorectal biopsy) and are provided to the pathologist only with a receipt number, with no organ distinction. Thus, the pathologist reads the slides as per the receipt number, with no organ distinction. In step 2, signed out slides are scanned using a scanner. WSIs generated by 3D HISTECH scanner have mrxs file extension and are saved in the directory designated by the scanner program. When a new WSI file is created in this directory, the Auto File Watcher program detects this and creates a copy in storage. In the SeeDP system, the scanned slide name (pathology number) is searched for in the DB, and the AI model corresponding to each organ is determined and implemented according to designed keywords in the heading (specimen information) of the pathology report (Table 3). The AI model reads the WSI file to perform slide-level and patch-level predictions and each outcome is saved in the DB. In step 3, using the SeeDP program, scanned slides are organized into classes by pathologic diagnosis and by AI prediction. Pathologists can access the SeeDP system the next day (or a few hours later) to check whether there is concordance between each class, and review discordant cases preferentially.

**Table 3:** Keywords to determine the appropriate AI model for each biopsy site.

AI models	Keywords
**Gastric**	Stomach, Esophagogastric junction, Gastroesophageal junction
**Colorectal**	Terminal ileum, Ileocecal valve, Cecum, Colon, Large intestine, Rectosigmoid junction, Colon and Rectum, Rectum

### Policies for the trial run of SeeDP for daily QC

#### Redefining classes for the SeeDP daily QC system

Between September 15, 2021 and December 14, 2021, four pathologists (YMC, JRK, BDL, and YSK) participated in the trial run. First, the classes of pathologic diagnoses were redefined as shown in Table 4.

**Table 4:** Definition of classes by the pathologic diagnosis for the SeeDP daily QC system.

Classes	Definition for daily QC
**M** *(Malignant)*	Malignancy or **high grade** dysplasia cannot be ruled out
**D** *(Dysplasia)*	**Low** grade dysplasia cannot be ruled out
**N** *(Negative for dysplasia)*	Negative for dysplasia (or worse)
**U** *(Uncategorized)*	**Neuroendocrine tumor (G1 or G2)** cannot be ruled out

**Abbreviations: QC** (quality control)

Classification for data training and validation in the AI model development phase (Table 2) and classification by the pathologic diagnosis in the trial run phase (Table 4) differed. Specifically, “tubular adenoma, high grade dysplasia (TA, HGD)” and “tubular adenoma, grade uncertain” were included in class D for training in the AI model development phase, but were redefined as class M in the SeeDP system for the daily QC trial run. Additionally, class D was redefined as low grade dysplasia (LGD), indefinite for dysplasia, and AUS. Each class was redefined in the trial run for clinical implementation to assist in the immediate correction of FNs by the rapid detection of potential human error by the pathologists, rather than to ensure a more accurate diagnosis by the AI model, as explained earlier. Therefore, specific words included in pathologic diagnoses were considered as keywords for classification by the pathologic diagnosis (Table 5).

**Table 5:** Keywords for classification by the pathologic diagnosis.

M *(Malignant)*		D *(Dysplasia)*	N *(Negative for dysplasia)*	U *(Uncategorized)*
Carcinoma	Sarcoma	Dysplasia	Sessile serrated adenoma/polyp	Neuroendocrine
High	Lymphoma	Adenoma	Sessile serrated adenoma	Carcinoid
Grade undetermined	Malignant	Low	Sessile serrated lesion	NET (Capital letters only)
Grade uncertain	Malignancy	Indefinite for dysplasia	Other (not M, D, U)	
Favor neoplastic	Cancer	Undetermined significance		

**Abbreviations:** NET (neuroendocrine tumor)

Based on the classification in Table 5, cases that included the corresponding keyword in the pathologic diagnosis (text information) were classified as the indicated class. Such classification results were compared (1:1 basis) against the classification by AI model prediction for determining concordance. Each keyword for classification was applied in the following order: M>U>D>N. However, “sessile serrated adenoma/polyp,” “sessile serrated adenoma,” and “sessile serrated lesion” were prioritized over the keywords for class D. For example, certain diagnoses, such as “Adenocarcinoma, moderately differentiated,” “Neoplastic lesion, suspicious for ***malignancy***,*”* “Tubular *adenoma*, ***high*** grade *dysplasia*,*” “*Tubulovillous adenoma, *low* to focal ***high*** grade *dysplasia*,*” “Adenoma* with focal ***carcinoma***tous change,” “Tubular *adenoma*, ***grade uncertain***,*” “****Malignant*** neoplasm,” *"*Atypical glandular proliferation, ***favor neoplastic***,” and “*Neuroendocrine* ***carcinoma***” involve italicized keywords, but are ultimately classified into class M based on the bolded keywords. Diagnoses such as “Tubular ***adenoma***, ***low*** grade ***dysplasia***,*” “*Atypical glandular proliferation, favor ***dysplasia***,” “Atypical glandular proliferation, ***indefinite for dysplasia***,” and “Atypical glandular proliferation, ***undetermined significance***” are classified as class D based on the rule described above. “Small cell nests with ***neuroendocrine*** feature” is also classified into class U, along with “***Neuroendocrine*** tumor, grade 1 (***carcinoid*** tumor).”

Based on the above-mentioned classification rule, if “Tubulovillous adenoma, *low* to focal ***high*** grade *dysplasia”* is considered as class M by pathologic diagnosis classification, but class D by WSI AI prediction, then this case is labeled as a “discordant” case, requiring review by a pathologist. Some ambiguous cases such as *"*Atypical glandular proliferation, ***favor neoplastic***,” “Atypical glandular proliferation, ***undetermined significance***,” and “Atypical glandular proliferation, ***indefinite for dysplasia***” could be classified into class M, D, and D, respectively, by pathologic diagnosis classification. However, the AI prediction result can be of class M, D, or even N, depending on the characteristics of each WSI. Such “non-typical” cases are also labeled as discordant with the AI prediction, requiring review by a pathologist.

For “non-typical” cases described above, additional slides (serial cut or recut slides) are often prepared. In such cases, the AI prediction results for each WSI may vary. For example, the prediction result for the original slide may be of class D, but one serial cut slide may be classified as class D, another slide may be classified as class D, and a deeper cut slide may be classified as class N, with disappearance of the lesion (D, D, M, and N, respectively). When AI predictions of serial slides are prepared from a single block and they vary like this, the final prediction was determined in the order of M>U>D>N. In the aforementioned example, the AI prediction would indicate “M” as the final prediction. If the pathologic diagnosis is “TA, LGD,” it would be marked as “D,” which would also be considered a discordant case. For simply improving concordance between the pathologic diagnosis and AI prediction (accuracy), “D,” which appeared most often (among D, D, M, and N) in the prediction results of each WSI slide, could be used as the final class. However, for post-analytic QC and the rapid detection of FN, the more clinically serious result was prioritized over the more frequent AI prediction result.

## Results

### Internal validation results

#### Performance in the laboratory (KAIST) validation

We trained the gastric and colorectal models using the methods described above. More specifically, those models were both based on a novel patch-based framework in which a WSI was split into multiple patches, the latent features from the patches were extracted for a patch-level classifier, and the patch-level features were aggregated for a slide-level classifier, both of which were based on DenseNet201. In laboratory tests, the WSI classifier achieved accuracy rates of 96.0% and 95.8% in classifying histopathological WSIs of gastric and colorectal endoscopic biopsy specimens, respectively, into three classes (Negative for dysplasia, Dysplasia, and Malignant). However, six WSIs were excluded from the assessment of the colorectal model due to errors. When these six WSIs were included, the accuracy dropped to 92.0%. Both models properly classified class N WSIs, but misclassified a small number of class D and M WSIs as class N (see S2 Table).

#### Performance in the in-house (SMF) validation

Before the real-world application of the finally selected two models, some of the daily signed-out endoscopic biopsy slides were used as a pre-test set for in-house validation. The pre-test was conducted using daily slides data generated in the real-world to verify the practicality of the application of the models in daily practice by indirectly testing the performance in clinical practice with unbalanced data distribution. We used 491 gastric biopsy WSIs and 319 colorectal biopsy WSIs in the pre-test. The percentage of class N is expected to be high in real-world practice; thus, this test was designed to verify whether the models can stably show high accuracy under similar conditions. Accordingly, while the lab validation data were evenly distributed by class, the pre-test dataset had a relatively higher number of class N WSIs. Consequently, gastric and colorectal models showed accuracies of 93.08% and 95.30%, respectively, which are suitable for the real-world implementation of daily QC (see S3 Table).

### Results of the SeeDP system for daily QC trial run

We developed and applied two separate AI models (gastric and colorectal models); however, pathologists do not need to consider each model separately in daily practice and they need to review only cases with discordance between the AI prediction and pathologic diagnosis. Accordingly, we first evaluated the overall performance instead of performance of each model. The performance of each model was then checked separately. The model that was finally applied was a ternary classifier model (classes M, D, and N); however, classification by pathologic diagnosis also included class U. Therefore, 16 WSIs (gastric biopsy: 2, colorectal biopsy: 14) corresponding to neuroendocrine tumor (NET) cases and belonging to class U, along with 53 WSIs considered as scan failures, were excluded from AI model performance assessment.

During the 3-month trial run period, the overall accuracy of the ternary classifier model was 90.29%, while the accuracies of the gastric and colorectal models were 89.81% and 90.81%, respectively (S4.1 Table in S4 Table). In particular, the negative predictive values (NPVs) of the overall, gastric, and colorectal models were high, i.e., 97.20%, 99.98%, and 92.99%, respectively. Therefore, if both the AI prediction and pathologic diagnosis were included in class N, additional verification for a FN seemed unnecessary. Accordingly, if pathologists diligently review discordant cases in accordance with the operating guidelines for the SeeDP daily QC system, FN pathology reports could be largely prevented. In particular, FN cases considered as severely discordant, classified as class M by pathologic diagnosis and class N by AI prediction, occurred in only one colorectal biopsy case (S4.1 Table in S4 Table). A closer examination of this case revealed that it was classified as class M based on a diagnosis of “TA, HGD”, but it was an ambiguous case with a small lesion size and possible interobserver discrepancy in dysplasia grading. The AI model also showed red heat at the patch level in the corresponding region, but the final prediction was class N (S7 Fig). In the gastric model, there were no serious FN cases that predicted class M as class N (S4.1 Table in S4 Table). Accordingly, it was determined that in our models, there was almost no probability of missing (classification as class N) “suspicious for malignancy” cases that a human pathologist might miss.

When each case was considered using a binary classification, class M (suspicious for malignancy) vs. non-M (no possibility of malignancy), the accuracies of the overall, gastric, and colorectal models were 97.36%, 96.22%, and 98.59%, respectively (S4.2 Table in S4 Table).

#### Distribution of the classification by AI prediction in the trial run for daily QC

In total, 26,133 WSIs were signed out by four pathologists and scanned during the 3-month period, comprising 13,630 gastric biopsy WSIs and 12,503 colorectal biopsy WSIs from 12,734 patients. Of these, there were 13 gastric biopsy WSIs and 40 colorectal biopsy WSIs that were not reviewed by the SeeDP daily QC system owing to scan failure (error) despite two or three re-scans. The distribution of AI predictions for specific pathologic diagnoses are shown in Tables 6 and 7. (The dataset of the present study are presented as S1 File).

**Table 6:** Distribution of the classification by AI prediction of gastric biopsy WSIs.

Pathologic diagnosis	M		D		N		Scan failed		Total (number, %)	
**Gastritis**										
CG	45	*0*.*96%*	299	*6*.*40%*	4322	*92*.*57%*	3	*0*.*06%*	4669	*34*.*26%*
CG + ero/u/reg/inflamed	265	*5*.*02%*	404	*7*.*65%*	4611	*87*.*28%*	3	*0*.*06%*	5283	*38*.*76%*
HCG	48	*5*.*06%*	31	*3*.*27%*	870	*91*.*68%*	-	*-*	949	*6*.*96%*
HCG + ero/u/reg/inflamed	73	*7*.*64%*	99	*10*.*37%*	782	*81*.*88%*	1	*0*.*10%*	955	*7*.*01%*
**Polyp (FGP, HP)**	57	*3*.*66%*	33	*2*.*12%*	1466	*94*.*16%*	1	*0*.*06%*	1557	*11*.*42%*
**Xanthoma/Xanthelasma**	1	*4*.*55%*	2	*9*.*09%*	19	*86*.*36%*	-	*-*	22	*0*.*16%*
**AUS/ Indefinite for dysplasia**	3	*23*.*08%*	8	*61*.*54%*	2	*15*.*38%*	-	*-*	13	*0*.*10%*
**LGD**	-	*-*	87	*98*.*86%*	1	*1*.*14%*	-	*-*	88	*0*.*65%*
**Dysplasia, grade uncertain**	1	*100*.*00%*	-	*-*	-	*-*	-	*-*	1	*0*.*01%*
**HGD, foveolar type**	-	*-*	1	*100*.*00%*	-	*-*	-	*-*	1	*0*.*01%*
**TA, HGD**	12	*57*.*14%*	9	*42*.*86%*	-	*-*	-	*-*	21	*0*.*15%*
**Atypical gl., favor neoplastic**	3	*100*.*00%*	-	*-*	-	*-*	-	*-*	3	*0*.*02%*
**Carcinoma**	38	*92*.*68%*	3	*7*.*32%*	-	*-*	-	*-*	41	*0*.*30%*
**Other**										
Inflammatory fibroid polyp	-	*-*	-	*-*	1	*100*.*00%*	-	*-*	1	*0*.*01%*
Russell body gastritis	1	*100*.*00%*	-	*-*	-	*-*	-	*-*	1	*0*.*01%*
Pyloric gland adenoma	1	*50*.*00%*	1	*50*.*00%*	-	*-*	-	*-*	2	*0*.*01%*
Malignant (type uncertain)	1	*100*.*00%*	-	*-*	-	*-*	-	*-*	1	*0*.*01%*
Neuroendocrine tumor	1	*50*.*00%*	-	*-*	1	*50*.*00%*	-	*-*	2	*0*.*01%*
s/f MALT lymphoma	-	*-*	-	*-*	1	*100*.*00%*	-	*-*	1	*0*.*01%*
Insufficient/foreign material	4	*21*.*05%*	-	*-*	10	*52*.*63%*	5	*26*.*32%*	19	*0*.*14%*
**Total (number. %)**	554	*4*.*06%*	977	*7*.*17%*	12086	*88*.*67%*	13	*0*.*10%*	**13630**	*100*.*00%*

**Abbreviations: CG** (chronic gastritis, chronic active gastritis), **ero** (erosion, erosive), **u** (ulcer, ulcerative), **reg** (regenerating, regenerative), **HCG** (*Helicobacter pylori* associated CG), **FGP** (fundic gland polyp), **HP** (hyperplastic polyp), **AUS** (atypical glandular proliferation, undetermined significant), **LGD** (low grade dysplasia), **HGD** (high grade dysplasia), **TA** (tubular adenoma), **gl.** (glands, glandular proliferation), **MALT** (mucosa-associated lymphoid tissue)

**Table 7:** Distribution of the classification by AI prediction of colorectal biopsy WSIs.

Pathologic diagnosis	M		D		N		Scan failed		Total (number, %)	
**Polyp**										
Hyperplastic	24	*0*.*36%*	323	*4*.*89%*	6250	*94*.*54%*	14	*0*.*21%*	6611	*52*.*88%*
Inflammatory	10	*2*.*95%*	29	*8*.*55%*	300	*88*.*50%*	-	*-*	339	*2*.*71%*
Lymphoid	1	*0*.*88%*	5	*4*.*39%*	108	*94*.*74%*	-	*-*	114	*0*.*91%*
**Inflammation**										
Nonspecific, inactive	5	*1*.*17%*	2	*0*.*47%*	419	*97*.*90%*	2	*0*.*47%*	428	*3*.*42%*
Active	21	*19*.*27%*	10	*9*.*17%*	77	*70*.*64%*	1	*0*.*92%*	109	*0*.*87%*
s/f IBD	2	*9*.*09%*	3	*13*.*64%*	17	*77*.*27%*	-	*-*	22	*0*.*18%*
**LGD**	102	*2*.*28%*	3812	*85*.*38%*	542	*12*.*14%*	9	*0*.*20%*	4465	*35*.*71%*
**focal HGD**	2	*40*.*00%*	3	*60*.*00%*	-	*-*	-	*-*	5	*0*.*04%*
**HGD**	15	*78*.*95%*	3	*15*.*79%*	1	*5*.*26%*	-	*-*	19	*0*.*15%*
**Carcinoma**	50	*98*.*04%*	1	*1*.*96%*	-	*-*	-	*-*	51	*0*.*41%*
**Other**										
Inflammatory fibroid polyp	-	*-*	-	*-*	2	*100*.*00%*	-	*-*	2	*0*.*02%*
Submucosal tumor	-	*-*	2	*9*.*52%*	19	*90*.*48%*	-	*-*	21	*0*.*17%*
SSL	-	*-*	38	*16*.*52%*	192	*83*.*48%*	-	*-*	230	*1*.*84%*
TSA	1	*2*.*17%*	26	*56*.*52%*	19	*41*.*30%*	-	*-*	46	*0*.*37%*
Neuroendocrine tumor	4	*28*.*57%*	-	*-*	10	*71*.*43%*	-	*-*	14	*0*.*11%*
Insufficient/foreign material	2	*7*.*41%*	-	*-*	11	*40*.*74%*	14	*51*.*85%*	27	*0*.*22%*
**Total (number, %)**	239	*1*.*91%*	4257	*34*.*05%*	7967	*63*.*72%*	40	*0*.*32%*	**12503**	*100*.*00%*

**Abbreviations: IBD** (inflammatory bowel disease), **LGD** (low grade dysplasia), **HGD** (high grade dysplasia), **SSL** (sessile serrated lesion, sessile serrated adenoma, sessile serrated adenoma/polyp), **TSA** (traditional serrated adenoma)

#### Analysis of cases discordant between the AI prediction and pathologic diagnosis

In both models, false positive (FP) rate was somewhat high. In the gastric model, the FP rate was higher for *H*. *Pylori*-associated chronic gastritis (HCG) than chronic gastritis among gastritis diagnoses, with an increased FP rate in cases with findings of a secondary reaction, related to erosion, ulcers, regenerative changes, and inflamed granulation tissue (+ ero/u/reg/inflamed) compared to that in cases without such findings (Table 6). In the colorectal model, cases of inflammatory polyps, active inflammation, or suspicious for inflammatory bowel disease showed a higher FP rate than nonspecific or inactive inflammation cases (Table 7). Moreover, such cases occasionally showed red heat in areas with inflammatory reactions or severe activity accompanied by erosion or ulceration in the patch-level heatmap (S8.1 Fig in S8 Fig). Such cases can be used as training data to improve the performance of future models when the pathologic diagnosis is found to be correct during the review of discordant cases by pathologists.

In the gastric model, there were some negative cases that were falsely predicted as class D. These cases showed blue heat in some areas, such as foveolar epithelium with some darker stained areas, poor quality sections with knife marks, (S8.2 Fig in S8 Fig) and intestinal metaplasia with atrophy. As shown as Table 6, two of these cases were xanthomatous lesions with no heat in the foamy histiocytes (S8.2 Fig in S8 Fig). However, in one case, some red heat was observed in an area accompanied by erosion; thus, it was incorrectly predicted as class M. In this area of the slide, the stroma was pressed by erosion, making it appear more cellular, and the cytoplasm was more pinkish than other areas; however, red heat was not observed in other areas with typical foamy histiocytes (S8.3 Fig in S8 Fig).

Ambiguous diagnoses, such as AUS, and “indefinite for dysplasia (IFD)”, were classified as class D by pathologic diagnosis and as class D by AI model prediction in 61.54% of such cases (8/13 cases). During model training, AUS and IFD initially belonged to class U in the quaternary model, but were excluded from the training dataset when the model was converted to a ternary model. However, it was very interesting to find that the accuracy was lower for AUS/IFD cases than for LGD despite the current model classifying most of them as class D. Moreover, "dysplasia, grade uncertain" and HGD belonged to class D when defining the training dataset (Table 2), but were redefined as class M by pathologic diagnosis when applied to the SeeDP system daily QC (Table 4). The gastric model tended to follow the redefined pathologic diagnosis classification, while the colorectal model tended to favor class D for focal HGD. However, for HGD cases, the colorectal model tended to follow the redefined classification, like the gastric model (Tables 6 and 7).

In the gastric model, one FN case, diagnosed as TA, LGD but classified as class N, had a small lesion, along with slight focal blue and red heat in portions of the WSI, which may have been neglected during spatial analysis in the WSI prediction step (S8.4 Fig in S8 Fig). This phenomenon was observed more frequently in the prediction of class D in the colorectal model than in the gastric model. The FN rate of LGD colorectal biopsies, predicted as class N, was 12.14%, which was higher than that in the gastric model (1.14%). However, owing to the difference in clinical significance between gastric LGD and colorectal LGD (gastric LGD requires more aggressive treatment), the class D prediction tendency of both models was considered to be practically ideal (Tables 6 and 7).

NET cases showed a tendency to be predicted as class N in both models, while the heatmap patterns mostly showed no heat (S8.5 Fig in S8 Fig). The four cases that were predicted as class M in the colorectal model were accompanied by unnecessary red heat associated with aforementioned inflammatory reaction. However, one specific case that was predicted as class M in the gastric model was somewhat different (S8.6 Fig in S8 Fig). While low-magnification findings seemed to show NET, high-magnification findings indicated that this case needed to be differentiated from “oxyntic gland adenoma exhibiting infiltrative growing pattern” and “gastric adenocarcinoma of the fundic-gland type”. This case was descriptively diagnosed as “favor NET,” for which the need for differentiation from these diagnoses, along with ancillary tests, were recommended. For such cases, it may be better to advise caution by classifying them as class M rather than as class N; thus, the response of this model was considered to be reasonable.

There was one case of s/f mucosa-associated lymphoid tissue (MALT) lymphoma within the daily QC set, which was predicted as class N with no heat in the WSI. The case involved HCG accompanied by lymphoid follicles (LFs), and localized lymphoepithelial body-like lesions were found near the LFs. The case was signed out with a descriptive diagnosis of Wotherspoon grade 3 (S8.7 Fig in S8 Fig). Although some FPs were occasionally found, which were predicated as class M with red heat in severe gastritis with LFs or lymphoid aggregate, in this case, a negative prediction was made.

### QC improvement status

Table 8 shows the comparison between QC performance in the three months of SeeDP daily QC system use in the trial run and the 33 months prior to the trial run. There was a total of 5,789 GI endoscopic biopsy slides that were randomly reviewed by monthly QC (monthly average of 160.8 slides) during the past three years (January 2019 to December 2021). Of these, there were 18 cases (0.31%) with discordance between the reviewer and the original diagnosis, comprising 16 mild (0.28%), two moderate (0.03%), and zero severe (0.00%) cases of discordance. There were no cases in which diagnostic errors identified by the monthly QC random review led to a revised diagnosis. Accordingly, we can claim that, at our institution, there were no cases in the past three years wherein the random review of QC slides ultimately affected the patient. There was a total of 26,080 slides that were double-checked by AI models during the 3-month SeeDP daily QC system trial run, of which, 814 slides were reviewed by human pathologists, in accordance with the operating plan.

**Table 8:** Comparison of detected diagnostic errors and corrections.

	Monthly QC only (33 m)	monthly QC + SeeDP daily QC (3 m)	Overall (36 m)
**Number of reviewed slides**	**5209**	**26660**	**31869**
via Random review (by 12–16 pathologists)	5209 (11.63/person/mo)	580 (12.08/person/mo)	5789
via AI model + 4 pathologists review	0	814 (67.83/person/mo)	}26080
via AI model only	0	25266	
**Detected discordance or diagnostic errors**	**29**	**13**	**42**
**via random review**	**18**	**0**	**18**
mild	16	0	16
moderate	2	0	2
severe	0	0	2
revision of Dx (time taken)	**0**	**0**	**0**
**via request from a clinician**	**6**	**1**	**7**
mild	4	1	5
moderate	2	0	2
severe	0	0	0
revision of Dx (time taken)	**6 (6.5 days)**	**0**	**6**
**via copy slide review**	**5**	**0**	**5**
mild	2	0	2
moderate	1	0	1
severe	2	0	2
revision of Dx (time taken)	**5 (80.6 days)**	**0**	**5**
**via previous bx review**	**0**	**4**	**4**
mild	0	0	0
moderate	0	3	3
severe	0	1	1
revision of Dx (time taken)	**0**	**0**	**0**
**via AI model + 4 pathologists review**	**0**	**8**	**8**
mild	0	5	5
moderate	0	2	2
severe	0	1	1
revision of Dx (time taken)	**0**	**8 (3.4 days)**	**8**
**Corrected or revised errors (time taken)**	**11 (40.2 days)**	**8 (3.4 days)**	**19**

**Abbreviations: QC** (quality control), **m** (month), **Dx** (diagnosis), **bx** (biopsy)

Among the GI endoscopic biopsy slides for the same 3-year period, there was a total of 16 cases that were processed as “discordant” or “error” after a non-random review. Of these, 11 cases were identified during the 33 months prior to the SeeDP daily QC system trial run and five cases occurred during the 3-month trial run period. Among the 11 pre-trial run cases, six cases involved a revised diagnosis based on a slide review requested by clinicians (four mild and two moderate cases of discordance). The remaining five cases involved a revised diagnosis based on the identification of additional lesions in newly prepared sign-out copy slides that were not found in the original slides (two mild, one moderate, and two severe cases of discordance). Of the five cases that were identified during the 3-month trial run period, four were diagnosed as negative in a previous biopsy (i.e., 6, 8, 10, or 16 months earlier), but the cases were reviewed during the interpretation of a follow-up biopsy, and previous diagnosis was determined to be a FN through internal communication. However, these cases were closed without revising the existing diagnosis because, according to the physicians who requested the test, it was too late to revise them, and these cases were managed based on the biopsy result currently in process (three moderate and one severe case of discordance; Table 8). The remaining eight cases (seven patients) were reviewed by both the AI model and pathologists; for these cases, the problem was recognized within an average of 1.2 days and the diagnosis was revised or corrected within an average of 3.4 days after communication with the physician who requested the test. In these cases, the errors were corrected before the patient received the pathology report from the clinician; thus, it can be considered that the daily QC system had a direct positive impact on these patients. In contrast, for cases reviewed by conventional methods, the diagnosis was revised or corrected only when it was unavoidable due to a sign-out of the copy slides and such measures took an average of 40.2 days.

The following are the specific details of cases that were corrected after detection through the SeeDP daily QC system (Table 9).

1. Detection and correction of FN cases
  - Major lesions missed in the microscopic FOV: In slides with two or more pieces of tissues, the pathologist missed a positive lesion in one piece in the microscopic FOV.
    - In rectal biopsy, one cancerous piece was missed in the FOV during microscopic interpretation; therefore, the case was initially diagnosed as negative. The SeeDP daily Qc system identified discordance between the AI prediction (M) and the pathologic diagnosis (D and N). Pathologists reviewed the WSIs and confirmed the presence of cancer, with red heat in the outermost piece. Based on this, the diagnosis was corrected (cases #1 and #2).
    - Similar to the above case, colon “TA, LGD”, missed in the FOV, was reviewed on the following day, whereby discordance between the AI prediction (D) and the pathologic diagnosis (N) was identified. Similarly, a lesion was confirmed in the WSI and heatmap; based on this, the diagnosis was corrected (case #3).
  - **Difficulty with diagnosis:** A case initially diagnosed as “CG with IM” during the routine practice of biopsy interpretation was classified as class D, with blue heat in the corresponding region, by AI prediction in the SeeDP system on the following day. Accordingly, the case was labeled as a discordant case (AI prediction of D versus pathologic diagnosis of N). The pathologist reviewed the WSI and glass slide and recalled the initial diagnosis after explaining the need for an additional intra-laboratory process to the hospital that had ordered the test. Serial and deeper slides were additionally prepared for testing and a second opinion from another pathologist was sought. Subsequently, the diagnosis was revised to “TA, LGD” (case #4).
  - Text diagnosis data entry error: A case labeled as “Resection margin: Free from tumor” without any major diagnosis in colon polypectomy was identified as a discordant case (AI prediction D versus pathologic diagnosis N). After a review of the WSIs, an error involving the omission of a “TA, LGD” diagnosis was detected and the diagnosis was subsequently corrected (case #5).
2. Detection and correction of FP cases
  Text diagnosis data entry error: In a negative biopsy case, a positive diagnosis was additionally entered by mistake during the data-entry stage. Although it was a colonic hyperplastic polyp (HP) case, two diagnoses (“HP” and “TA, LGD”) were inadvertently entered. The case was marked as class D in accordance with the pathologic diagnosis classification rules, but AI prediction classified the case as class N because the corresponding lesion was not found on the WSI. Accordingly, the case was labeled as a discordant case. Following the daily review procedure, a pathologist reviewed the applicable WSIs and recognized the error of a positive diagnosis entry; based on this, the diagnosis was corrected (case #6).
  Sometimes, a colorectal biopsy of two or more pieces may actually show the co-presence of “TA, LGD” and “HP,” and in such cases, the two diagnoses are entered together for each piece. Consequently, it is difficult to confirm an error based solely on the entry of two diagnoses. However, this type of error was detected when the AI model reviewed it based on the WSIs to assess concordance with the diagnostic data.
3. Detection and correction of a switching error
  Erroneous switching of paired specimens: Cecal and sigmoid colon biopsy specimens collected from the same patient were received with the slides in reverse order, and the pathologist read the slides without recognizing this error. One slide was diagnosed as “TA, LGD” and the other slide was diagnosed as “nonspecific inflammation.” However, because the slides were processed by the QR codes that appear on the slide label in the AI-assisted SeeDP daily QC system, each slide was predicted as “N and D” based on the WSIs, which was in discordance with the pathologic diagnosis of class “D and N.” Accordingly, a pathologist reviewed the slides the following day, in accordance with the daily QC system operating procedure, and identified the error. Subsequently, the hospital that ordered the test was notified and the diagnosis was corrected (case #7).

**Table 9:** Details of seven cases of error identified with the SeeDP daily QC system.

Case number	Type of error	Class by		Initial pathologic Dx	Revised Dx	Time taken (days)
		1’ Dx	AI			
1	**FN**	**D**	**M**	Rectum, colonoscopic biopsy: **TA, LGD**	Rectum, colonoscopic biopsy: **ADENOCARCINOMA, MD**	**2**
2	**FN**	**N**	**M**	Rectum, colonoscopic biopsy: **Focal active proctitis**	Rectum, colonoscopic biopsy: **ADENOCARCINOMA, MD**	**1**
3	**FN**	N	D	Rectum, colonoscopic biopsy: Hyperplastic polyp	Rectum, colonoscopic biopsy: A. TA, LGD (x1) B. Hyperplastic polyp (x1)	8
4	**FN**	N	D	Stomach, endoscopic biopsy: CG with IM	Stomach, endoscopic biopsy: Atypical gland proliferation, **favor TA, LGD**	3
5	**FN**	N	D	Ileocecal valve, colonoscopic polypectomy: • Resection margin: Free from tumor	Ileocecal valve, colonoscopic polypectomy: **TA, LGD** • Resection margin: Free from tumor	1
6	**FP**	D	N	Rectum, colonoscopic biopsy: Hyperplastic polyp **TA, LGD**	Rectum, colonoscopic biopsy: Hyperplastic polyp	8
7	**Switching**	D N	N D	01. Cecum, colonoscopic mucosal resection: **TA, LGD** • Resection margin: Uncheckable 02. Colon, colonoscopic mucosal resection: Hyperplastic polyp	01. Cecum, colonoscopic mucosal resection: Hyperplastic polyp 02. Colon, colonoscopic mucosal resection: **TA, LGD** • Resection margin: Uncheckable	2

**Abbreviations: QC** (quality control), **Dx** (diagnosis), **FN** (false negative), **MD** (moderately differentiated), **TA** (tubular adenoma), **LGD** (low grade dysplasia), **CG** (chronic gastritis), **IM** (intestinal metaplasia), **FP** (false positive)

## Discussion

### A clinically applicable AI model and DP total solution

#### Our system was designed so that AI models could be actively used without excess change to the conventional workflow of the diagnostic process

We opted for a method that could quickly detect human errors that may occur during pathologic interpretation, allowing immediate corrections without affecting the actual interpretation process. In the daily practice of pathologists, GI endoscopic biopsy slides are provided without distinction between gastric and colorectal biopsy specimens; and are interpreted according to their serial number. In many cases, gastric and colorectal biopsy specimens from the same patient are received and processed consecutively; it is not suitable to process such slides by differentiating them according to organ. However, the SeeDP program is designed to selectively run the AI model corresponding to each organ based on the organ information listed in the heading of each specimen. Therefore, when pathologists review slides through the AI-assisted daily QC system, they can review them by serial number as well. Additionally, when pathologists perform diagnoses through WSIs and want to refer to the prediction results of the AI model, they can follow the receipt number.

However, if the heading information and the organ do not match, the model designed for that organ cannot be run without revising the heading. Moreover, the models cannot be run for other organs that may be present together in the GI endoscopic biopsy specimens, such as the esophagus, duodenum, and anus. Furthermore, for various diseases belonging to class U (e.g., NET, MALT, submucosal tumor, inflammatory bowel disease, and serrated lesions), which were initially excluded from the study, our current AI models do not yet provide a cautionary warning. Therefore, further research is needed to overcome this limitation (i.e. including other diseases). Therefore, we are currently investigating an upgrade to a quaternary classifier model that would include NET grade 1 or 2.

#### Our goal was 100% practical application

The results of our study were obtained by scanning all microscopic slides signed out daily and running AI models, instead of simply selecting interesting cases for assistance with AI models. We aimed to develop AI models for all GI endoscopic biopsy specimens to achieve 100% practical application in daily QC. During the trial run period, all endoscopic biopsy slides signed out by four pathologists were 100% digitalized and almost all were double checked by the AI models. However, the system is still not applicable for non-endoscopic biopsy and cytology specimens. Additional research and development efforts are needed for other organs and all specimens received for testing.

#### We developed our own post-analytic daily QC system with an AI-assisted DP total solution (SeeDP) that integrates a WSI viewer and AI models, combined with a pathology LIS

First, for the early detection of potential human errors in microscopic interpretations based on conventional methods, we designed a system to differentiate specimens based on the heading information in the pathologic report and automatically labelling and applying either the gastric or colorectal model, as suitable for that specimen. Next, we set major keywords in the text information of the pathologic diagnosis for automatic classification of cases into three classes (classes M, D, and N). Moreover, the prediction results from the AI model suitable for each specimen were exposed to the text classification of M, D, and N for comparison against classification by pathologic diagnosis to check for concordance. Discordant cases were listed first, so that pathologists could prioritize their review. When the pathologists reviewed these cases, the AI prediction results were not visualized by heatmaps provided separately from WSIs; rather, the heatmaps were masked on or off directly on top of WSIs to enable an intuitive comparison between the WSI and heatmap at all magnifications. Lastly, we included functions that enabled pathologists to assess the AI prediction results for reviewed cases and leave their feedback. By continuing to collect FN and FP cases and using them as an additional model training resources, we were able to establish a system with continuous improvement in model performance.

However, the system cannot detect cases in which both the AI prediction and pathologic diagnosis are FNs. Therefore, the models we developed and implemented were designed to maintain high NPV to minimize such risk.

### A clinically influential AI-assisted daily QC system

#### We developed a powerful and clinically influential system that can ultimately help enhance patient safety

Numerous researchers have contemplated and suggested improvements in, and the standardization of, test methods that can clinically enhance patient safety. In particular, in the field of gastroenterology, an endoscopy is performed first; if necessary, a tissue biopsy is performed to obtain a confirmative diagnosis through a histopathological examination. In this process, a discrepancy may exist between the endoscopic findings and the histopathologic diagnosis, and patient safety is not assured given this inconsistency. Therefore, studies to enhance the patient’s stability from the stage prior to biopsy have been continuously conducted, and recent studies using AI are noteworthy [54, 55]. Similarly, pathologic laboratory QC ensures high reliability in the quality of testing, which ultimately helps in enhancing patient safety [10]. The monthly review of random slides, which is the most popular method among conventional post-analytic QC methods, serves an important internal function for narrowing the discrepancies between pathologists by blinded reviews, communication of discordant cases, and regular sharing of feedback [9]. In many cases, however, reviewers tend to ignore or dismiss minor discrepancies in existing diagnoses and in their approach to the review process. This may be because the time gap between the original diagnosis and review is at least one month or longer. Therefore, although the random slide review may be a key internal function, it often does not have direct clinical influence on patients or the clinicians ordering the test. Additionally, 1–2 months may have already passed since the initial diagnosis of a severe discordance. Therefore, even if the corrected diagnosis is conveyed to the clinician or the patient, the previous FN may have deprived the patient of appropriate treatment.

Improvements in pathology laboratory QC achieved by combining our AI-assisted daily QC system with a monthly review of random slides can be easily inferred through the comparisons in Table 8. In summary, the currently used monthly random slide reviews, performed by an average of 14 pathologists during the 33 months prior to the SeeDP AI-assisted daily QC system trial run had relatively little practical influence on detecting or correcting hidden errors in pathologic diagnoses. In contrast, 11 hidden errors were corrected within an average of 40.2 days by slide reviews compared to the methods used during that 33-month period. Further, eight cases of errors were detected within 1.2 days by four pathologists during the 3-month SeeDP AI-assisted daily QC system trial run period, and corrections were made within an average of 3.4 days. As a comparison, when the duration and number of human pathologists were roughly adjusted based on these results, we found that approximately 7–10 times as many slides could be reviewed by pathologists, compared with conventional methods and nearly 100% of GI endoscopy slides can be double-checked by the AI models; approximately 17–30 times as many current potential human errors could be detected within an average of 1.2 days. Consequently, more active measures could be expected before the erroneous pathology report is conveyed to the patient. These findings indicate that the AI-assisted daily QC system is a direct and powerful tool for improving daily QC, in quantitative, qualitative, and time utility aspects, while also being clinically influential, from a patient safety aspect.

As seen in Table 9, errors detected and corrected by the AI models were mostly human errors, rather than those caused by the diagnostic ability of the pathologist or the difficulty of the diagnosis itself. Such potential human errors are often difficult to recognize by medical record officers or pathologists by simply reviewing the diagnostic information, especially when lesions are missed in the microscopic FOV or when an incorrect diagnosis is made without typographical errors. Many studies have shown that AI models can improve the diagnostic accuracy of cases that are difficult to diagnose, and have a positive impact on junior pathologists and trainees [29, 56]. However, they do not provide substantive assistance to pathologists with sufficient experience. Additionally, because the concept of ambiguity always exists in the field of pathology, many pathologists worldwide are continuously endeavoring to reduce discrepancies through periodic discussions and agreements; this problem may be an everlasting obstacle for pathologists to overcome. Further, most pathologists try to perform an accurate diagnosis (or minimize the possibility of a misdiagnosis) when they encounter cases that are considered difficult to diagnose. Some of the actions pathologists take for patients (and for themselves too) include preparing recut or serial cuts, ordering ancillary tests, referring to past slides, thoroughly reviewing medical records, seeking secondary opinions or advice from experts, reviewing similar cases, and looking up related literature. It is doubtful that the AI model could considerably replace the diagnostic capabilities of pathologists compared to the result of the previously mentioned accumulated efforts. The real problem may be when pathologists sign out in daily practice without realizing the possibility of making mistakes. As discussed above, unpredictable simple human errors ultimately result in errors that are difficult to detect in conventional monthly random reviews. A lot of such hidden human errors, like those mentioned above have likely persisted, and some of these hidden errors can baffle pathologists months or years later. Moreover, patients may have to face unnecessary burdens.

### Our system has certain limitations, but we have plans to incrementally make our system more robust

AI systems are onerous to utilize with the emergence of data with new characteristics. For example, if detection is required for a minimum number of lesions (a cause of FNs), it is expected that the proposed model will have difficulty in responding to the request because the model is operated based on spatial information. It is also necessary to strengthen the ability to accurately categorize a small number of histological properties that cause FPs, including some tissue artifacts and atypical reactive changes. Although relearning these specific cases is expected to improve performance, there are practical difficulties in preparing training data for all peculiar cases. Therefore, as in previous studies, methods for continuous model updates based on active learning can be used for continuous DNN learning, as an alternative measure [57–59].

Additionally, impact analysis on various factors needs to be conducted to establish a general-purpose AI system that can be used by external organizations. Slightly different images can be produced even if the same scanner from the same company is used. Therefore, it is necessary to develop robust models that can produce consistent performance by collecting data on various cases, including the color heterogeneity problem, which is widely considered, and conducting an impact analysis on various factors.

Although the proposed classifier models exhibit appropriate functions and numerous contributions within the actual QC system, we aimed to produce more enhanced classifier models in further research. Approaches from two main perspectives are deemed necessary to improve the performance of the proposed models, with the first concerning the data training perspective. In the present study, we observed that the prediction capabilities of the model decreased for some cases with the expansion of data. This could be due to the typical limitations of DNN-based classification models, i.e., they cannot respond well to cases outside the range of the learned data. However, the model needs to respond to more diverse cases to enable its usage in practice. This particular problem can be mitigated through the expansion of learned data. A typical alternative involves producing a consistent training system based on active learning to induce consistent learning in the classifier model, updating a wider range of data. The second perspective concerns performance enhancement from the model architecture perspective. Although the present study actively utilized well-known CNN-based architecture, novel approaches have been recently investigated to overcome the limitations of CNNs. A typical example is the Vision Transformer (ViT), which is actively used in various imaging studies [60, 61]. The ViT model has a more simplified structure than the CNN architecture, and it mainly utilizes regional information. This simplified structure enables the implementation of operations by minimizing the loss of information in the entire image. The performances of patch classifiers and WSI classifier models themselves can be improved through the active utilization of models such as ViT, which can overcome the limitations of CNNs.

### Significance of our study and suggestions regarding our system

The limitations of DP prevent the complete replacement of the conventional pathology methods of reviewing glass slides through a microscope. However, technological developments will enable a continuous increase in device performance, gradual reduction of infrastructure construction costs, and vitalization of the development of WSI or DP-based AI models and software. In future, all pathologists will choose WSI as the primary diagnostic tool, and they will rely on computation software for more complex and significant tasks, completely replacing microscopes in the pathologists’ office with 100% DP. Thus, it may be better to use the AI models developed in the present study as pre-analytic screening tools, rather than as post-analytic QC tools. The AI models could provide an excellent AI-assisted pathology solution that can terminate the inspection of screened negative cases, without the confirmation of a human pathologist. However, we do not have to wait for this level of high-end technology to completely change our work life because we already possess technology that can be applied immediately to our daily work and is useful to pathologists, clinicians, and patients. When the purpose of utilization was slightly altered, we confirmed that its application to real-world clinical practice became possible. There have been studies on pre-analytic QC AI models that have attempted to apply AI as a QC tool [35, 62]. Each focused on the QC of the WSI scan quality itself, or to enhance the performance of the AI models. However, this was the first large-scale study wherein an AI model was developed, applied, and operated as a daily fast QC tool in the post-analytic phase for the enhancement of pathologic diagnosis quality management by quickly detecting and responding to potential human errors and ultimately enhancing patient safety.

In conclusion, we developed AI models with reliable performance and applied them as post-analytic daily fast QC tools in the routine practice of GI endoscopic interpretation. The AI-assisted daily QC system that we developed and established demonstrated notable improvements in QC, in quantitative, qualitative, and time utility aspects. Ultimately, we developed an independent AI-assisted post-analytic daily QC system that was clinically applicable and influential, which could enhance patient safety.

## Supporting information

S1 Methods Annotation rules. (DOCX)

S1 Table Major equipment and specifications. (DOCX)

S2 Table Accuracy of the developed models (KAIST laboratory validation test). (DOCX)

S3 Table Accuracy of the developed models (SMF in-house validation). (DOCX)

S4 Table S4.1 Table. Ternary classifier AI model performance. S4.2 Table. Binary classification AI model performance.
(ZIP)

S1 Fig Display of an example “Test Results” page in the SeeDP system. In the SeeDP system, scanned slide data can be searched by receipt date, inspection date, and scan date on the “Test Results” and “Statistics” pages. The “Test Results” page provides the slide information list for each WSI, including the receipt date, inspection date, pathology number (slide name), patient name, classification by pathologic diagnosis, classification by AI prediction, concordance, AI model (anatomy), and pathologist (reader). It also provides text information, including a AI model heatmap thumbnail at single magnification (0.5x), pathologic diagnosis, notes, and previous pathologic diagnoses. **Abbreviations:** AI (artificial intelligence), SeeDP (Seegene Medical Foundation’s AI-assisted Digital Pathology Total Solution), WSI (whole slide image).
(DOCX)

S2 Fig An example of the “Statistics” page in the SeeDP system. From the “Statistics” page, the prediction performance and distribution of each AI model (gastric/colorectal) can be checked. Moreover, the pathologist can review the applicable WSIs by selecting the cells within the “AI Distribution” table, which allows the pathologist to selectively review the cases that meet desired conditions from the “Statistics” page, similar to the “Test Results” page. **Abbreviations:** AI (artificial intelligence), SeeDP (Seegene Medical Foundation’s AI-assisted Digital Pathology Total Solution), WSI (whole slide image).
(DOCX)

S3 Fig Display of the WSI viewer, zoomed in and out. The viewer allows 3DHISTECH WSI files in mrxs file format to be loaded directly; it has a fast image loading speed and excellent scalability because it can be installed in a desktop computer as well as a laptop, mobile phone, or tablet. When the slides list is searched in the SeeDP system and the row of a specific slide is double clicked, the WSI viewer for that slide appears. The image location can be moved by dragging the screen, and the mouse scroll button can be used to zoom in/out, while the mini-map in the upper right corner can be used to identify the location displayed on the screen. **Abbreviations:** WSI (whole slide image).
(DOCX)

S4 Fig Display of serial-section WSIs. When there are multiple slides from a single specimen (presence of a recut, serial, or deeper section, or the presence of more than two blocks), the WSI viewer provides a related slide list on the upper left corner to view related WSIs together. **Abbreviations:** WSI (whole slide image).
(DOCX)

S5 Fig S5.1 Fig. Menu buttons. The function buttons in the lower left corner can be used to run various WSI-related functions. From the left, the functions are as follows: perform rotation, visualization of the AI heatmap and prediction, split the screen, position movement, measurement, annotation insertion, annotation lookup, annotation storage, and annotation deletion functions. Abbreviations: AI (artificial intelligence), WSI (whole slide image). S5.2 Fig. Rotation of the whole slide image. S5.3 Fig. Split screen. S5.4 Fig. Annotation.
(ZIP)

S6 Fig WSI viewer–Visualization of the heatmap and prediction of the AI model. We developed a visualization function that would allow slide-level and patch-level information to be viewed on top of a single WSI in the WSI viewer. The final slide-level prediction is marked with a text label on the bottom of the mini-map in the upper right corner of the WSI viewer. Moreover, patch-prediction information is produced in heatmap format, and based on location information for each patch, prediction information is expressed on top of each patch within the WSI. Class M, D, and N patches are expressed as red, blue, and no heat. In particular, heatmap mask-on and -off can be enabled by simply right-clicking while viewing the slides through the WSI viewer. The basic functions of the WSI viewer, such as zoon in/out and rotation, can be used to simultaneously view the WSI and heatmap at all magnifications (0.5x–40x). Accordingly, when a human pathologist reviews WSIs, he or she can intuitively check how AI models inferred the parts of a single WSI while turning the heatmap on and off at all magnifications. **Abbreviations:** AI (artificial intelligence), WSI (whole slide image), M (Malignant), D (Dysplasia), N (Negative for dysplasia).
(DOCX)

S7 Fig WSI and heatmap of the only case of severe discordance. This case was classified as class M by pathologic diagnosis and class N by AI prediction, and was only case of severe discordance. It was revealed that this case was classified as class M based on a diagnosis of “TA, HGD”, but it was an ambiguous case with small lesion size and possible interobserver discrepancy in the dysplasia grading. The AI model showed red heat at the patch-level in the corresponding region, but the final prediction was class N. **Abbreviations:** AI (artificial intelligence), WSI (whole slide image), M (Malignant), N (Negative for dysplasia), TA (tubular adenoma), HGD (high grade dysplasia).
(DOCX)

S8 Fig S8.1 Fig. A representative false positive case (N to M) in the gastric model. Class M prediction with red heat on the ulcer-related change areas. This case was diagnosed as HCG with ulcer. Abbreviations: M (Malignant), N (Negative for dysplasia), TA (tubular adenoma), HCG (H. Pylori-associated chronic gastritis). S8.2 Fig. A representative false positive case (N to D) in the gastric model. Class D prediction with blue heat on the darkly stained and tangentially sectioned foveolar epithelium with knife marks. No heat in the other xanthomatous areas. This case was diagnosed as xanthoma. Abbreviations: D (Dysplasia), N (Negative for dysplasia). S8.3 Fig. A representative false positive case (N to M) in the gastric model. Class M prediction with red heat in erosion-related change areas. This case was diagnosed as xanthelasma with erosion. There was no heat in other xanthomatous areas. Abbreviations: M (Malignant), N (Negative for dysplasia). S8.4 Fig. The only false negative case (D to N) in the gastric model. Class N prediction with focal blue and red heat only in the dysplastic area. This case was diagnosed as TA, LGD. Abbreviations: D (Dysplasia), N (Negative for dysplasia), TA (tubular adenoma), LGD (low grade dysplasia). S8.5 Fig. Representative cases for NET of the stomach and colon. Most of these cases were predicted as class N with no heat in both models. Abbreviations: N (Negative for dysplasia), NET (neuroendocrine tumor). S8.6 Fig. Histopathologic findings of a specific case diagnosed as “favor NET” and predicted as class M in the gastric AI model. While low-magnification findings seemed to show NET, high-magnification findings indicated that this case needed to be differentiated from “oxyntic gland adenoma exhibiting infiltrative growing pattern” and “gastric adenocarcinoma of the fundic-gland type”. Abbreviations: AI (artificial intelligence), M (Malignant), NET (neuroendocrine tumor). S8.7 Fig. Histopathologic findings of a specific case of “s/f MALT lymphoma” and predicted as class N in the gastric AI model. This case involved HCG accompanied by lymphoid follicles. Localized lymphoepithelial body-like lesions were found near the lymphoid follicles. The case was signed out with a descriptive diagnosis of Wotherspoon grade 3. Abbreviations: AI (artificial intelligence), N (Negative for dysplasia), MALT (mucosa-associated lymphoid tissue), HCG (H. Pylori-associated chronic gastritis).
(ZIP)

S1 File Raw data from Tables 6 and 7. (XLSX)
