# Supplementary material for: Improving quality control in the routine practice for histopathological interpretation of gastrointestinal endoscopic biopsies using artificial intelligence
Source: PLoS One. 2022 Dec 15;17(12):e0278542. doi: 10.1371/journal.pone.0278542 (PMC9754254; doi:10.1371/journal.pone.0278542)
Supplement: S1 Methods — (DOCX) [file pone.0278542.s001.docx]

**S1 Methods. Annotation rules**

Whole slide images (WSIs) in each class were reviewed and annotated by pathologists with at least five years of clinical experience. Using the CaseViwer program from 3DHISTECH, each pathologist independently made manual annotations. A single closed curve was drawn, with red line for class M and blue line for class D. If the boundary of the lesion was ambiguous, an outline was drawn to include only definite lesions (in both the epithelium and stroma). Only the patches within a single closed curve were used as data for each class; all other parts were removed. For class N, the pathologists reviewed and confirmed the WSIs according to the above definition, and they were used as data after generating patches without separate annotations (areas other than the lesions in class M and D slides were not used as class N data). We did not include non-neoplastic glands or crypts within the single closed curve; we also attempted to exclude non-tissue components, such as necrosis, ulcer detritus, extracellular mucin pool, and blood. Annotations based on these rules required an average of approximately 10–15 minutes for each WSI. When necessary, annotations were reviewed by another pathologist and revised or excluded accordingly.
