# Supplementary material for: Improving quality control in the routine practice for histopathological interpretation of gastrointestinal endoscopic biopsies using artificial intelligence
Source: PLoS One. 2022 Dec 15;17(12):e0278542. doi: 10.1371/journal.pone.0278542 (PMC9754254; doi:10.1371/journal.pone.0278542)
Supplement: S1 Table — (DOCX) [file pone.0278542.s002.docx]

**S1 Table. Major equipment and specifications**

| **Scanner** | **Name** | 3D HISTECH - Pannoramic 250 flash 3 (x3) |
| --- | --- | --- |
| **Digital Pathology System Server** | **OS** | Windows Server 2019 standard |
|  | **Process** | Intel(R) Xeon® Gold 5217 CPU @3.00 GHz (x2) |
|  | **Memory** | 512 GB |
|  | **Storage** | VAST Data Storage 450 TB |
|  | **Database** | MariaDB 10.4 |
| **GPU Server** | **OS** | Ubuntu 20.04.3 LTS |
|  | **Process** | Intel(R) Xeon® Gold 6330 Processor (x2) |
|  | **Memory** | 3200 MHz ECC DDR4 RDIMM 16 GB |
|  | **GPU** | GPU Nvidia A100 40 GB Pcie (x2) |

**Abbreviations:** OS (operating system), GPU (graphics processing unit)
