# Supplementary material for: Improving quality control in the routine practice for histopathological interpretation of gastrointestinal endoscopic biopsies using artificial intelligence
Source: PLoS One. 2022 Dec 15;17(12):e0278542. doi: 10.1371/journal.pone.0278542 (PMC9754254; doi:10.1371/journal.pone.0278542)
Supplement: S2 Table — (DOCX) [file pone.0278542.s003.docx]

**S2 Table. Accuracy of the developed models (KAIST laboratory validation test)**

| **AI model prediction** | **Gastric** | | | | **Colorectal** | | | |
| --- | --- | --- | --- | --- | --- | --- | --- | --- |
|  | **Classes** | | | **Sum** | **Classes** | | | **Sum** |
|  | **M** | **D** | **N** |  | **M** | **D** | **N** |  |
| **M** | 46 | 0 | 0 | 46 | 49 | 1 | 0 | 50 |
| **D** | 1 | 48 | 0 | 49 | 1 | 39 | 0 | 40 |
| **N** | 3 | 2 | 50 | 55 | 0 | 4 | 50 | 54 |
| **Sum** | 50 | 50 | 50 | 150 | 50 | 44 (6e) | 50 | 144 |
| **Accuracy** | **96.00 %** | | | | - 1. **(92.0) %** | | | |

**Abbreviations:** AI (artificial intelligence), M (Malignant), D (Dysplasia), N (Negative for dysplasia)
