# Supplementary material for: Improving quality control in the routine practice for histopathological interpretation of gastrointestinal endoscopic biopsies using artificial intelligence
Source: PLoS One. 2022 Dec 15;17(12):e0278542. doi: 10.1371/journal.pone.0278542 (PMC9754254; doi:10.1371/journal.pone.0278542)
Supplement: S3 Table — (DOCX) [file pone.0278542.s004.docx]

**S3 Table. Accuracy of the developed models (SMF in-house validation)**

| **AI model prediction** | **Gastric** | | | | **Colorectal** | | | |
| --- | --- | --- | --- | --- | --- | --- | --- | --- |
|  | **Classes** | | | **Sum** | **Classes** | | | **Sum** |
|  | **M** | **D** | **N** |  | **M** | **D** | **N** |  |
| **M** | 97 | 0 | 8 | 105 | 84 | 1 | 0 | 85 |
| **D** | 4 | 144 | 10 | 158 | 0 | 80 | 1 | 81 |
| **N** | 10 | 2 | 216 | 228 | 0 | 13 | 140 | 153 |
| **Sum** | 111 | 146 | 234 | 491 | 84 | 94 | 141 | 319 |
| **Accuracy** | **93.08 %** | | | | - 1. **%** | | | |

**Abbreviations:** AI (artificial intelligence), M (Malignant), D (Dysplasia), N (Negative for dysplasia), SMF (Seegene Medical Foundation)
