# Supplementary material for: Improving quality control in the routine practice for histopathological interpretation of gastrointestinal endoscopic biopsies using artificial intelligence
Source: PLoS One. 2022 Dec 15;17(12):e0278542. doi: 10.1371/journal.pone.0278542 (PMC9754254; doi:10.1371/journal.pone.0278542)
Supplement: S4 Table — S4.1 Table. Ternary classifier AI model performance. S4.2 Table. Binary classification AI model performance. (ZIP) [file pone.0278542.s005.zip › S4.1 Table.docx]

**S4.1 Table. Ternary classifier AI model performance**

| **Overall** | | | **Pathologic diagnosis** | | | **Sum** |
| --- | --- | --- | --- | --- | --- | --- |
|  |  |  | **M** | **D** | **N** |  |
| **AI Prediction** | | **M** | 120 | 105 | 563 | 788 |
|  |  | **D** | 20 | 3932 | 1282 | 5234 |
|  |  | **N** | 1 | 560 | 19481 | 20042 |
| **Sum** | | | 141 | 4597 | 21326 | **26064** |
| **Accuracy** | | | **90.29%** | | |  |
| **Gastric** | | | **Pathologic diagnosis** | | | **Sum** |
|  |  |  | **M** | **D** | **N** |  |
| **AI Prediction** | | **M** | 52 | 3 | 498 | 553 |
|  |  | **D** | 13 | 94 | 870 | 977 |
|  |  | **N** | 0 | 3 | 12082 | 12085 |
| **Sum** | | | 65 | 100 | 13450 | **13615** |
| **Accuracy** | | | **89.81%** | | |  |
| **Colorectal** | | | **Pathologic diagnosis** | | | **Sum** |
|  |  |  | **M** | **D** | **N** |  |
| **AI Prediction** | **M** | | 68 | 102 | 65 | 235 |
|  | **D** | | 7 | 3838 | 412 | 4257 |
|  | **N** | | 1 | 557 | 7399 | 7957 |
| **Sum** | | | 76 | 4497 | 7876 | **12449** |
| **Accuracy** | | | **90.81%** | | |  |

**Abbreviations:** AI (artificial intelligence), M (Malignant), D (Dysplasia), N (Negative for dysplasia)
