# Supplementary material for: Improving quality control in the routine practice for histopathological interpretation of gastrointestinal endoscopic biopsies using artificial intelligence
Source: PLoS One. 2022 Dec 15;17(12):e0278542. doi: 10.1371/journal.pone.0278542 (PMC9754254; doi:10.1371/journal.pone.0278542)
Supplement: S4 Table — S4.1 Table. Ternary classifier AI model performance. S4.2 Table. Binary classification AI model performance. (ZIP) [file pone.0278542.s005.zip › S4.2 Table.docx]

**S4.2 Table. Binary classification AI model performance**

| **Overall** | | **Pathologic diagnosis** | | **Sum** |
| --- | --- | --- | --- | --- |
|  |  | **M** | **non-M** |  |
| **AI Prediction** | **M** | 120 | 668 | 788 |
|  | **non-M** | 21 | 25255 | 25276 |
| **Sum** | | 141 | 25923 | **26064** |
| **Accuracy** | | **97.36%** | |  |
| **Gastric** | | **Pathologic diagnosis** | | **Sum** |
|  |  | **M** | **non-M** |  |
| **AI Prediction** | **M** | 52 | 501 | 553 |
|  | **non-M** | 13 | 13049 | 13062 |
| **Sum** | | 65 | 13550 | **13615** |
| **Accuracy** | | **96.22%** | |  |
| **Colorectal** | | **Pathologic diagnosis** | | **Sum** |
|  |  | **M** | **non-M** |  |
| **AI Prediction** | **M** | 68 | 167 | 235 |
|  | **non-M** | 8 | 12206 | 12214 |
| **Sum** | | 76 | 12373 | **12449** |
| **Accuracy** | | **98.59%** | |  |

**Abbreviations:** AI (artificial intelligence), M (Malignant)
