# Supplementary material for: Improving quality control in the routine practice for histopathological interpretation of gastrointestinal endoscopic biopsies using artificial intelligence
Source: PLoS One. 2022 Dec 15;17(12):e0278542. doi: 10.1371/journal.pone.0278542 (PMC9754254; doi:10.1371/journal.pone.0278542)
Supplement: S1 Fig — In the SeeDP system, scanned slide data can be searched by receipt date, inspection date, and scan date on the “Test Results” and “Statistics” pages. The “Test Results” page provides the slide information list for each WSI, including the receipt date, inspection date, pathology number (slide name), patient name, classification by pathologic diagnosis, classification by AI prediction, concordance, AI model (anatomy), and pathologist (reader). It also provides text information, including a AI model heatmap thumbnail at single magnification (0.5x), pathologic diagnosis, notes, and previous pathologic diagnoses. Abbreviations: AI (artificial intelligence), SeeDP (Seegene Medical Foundation’s AI-assisted Digital Pathology Total Solution), WSI (whole slide image). (DOCX) [file pone.0278542.s006.docx]

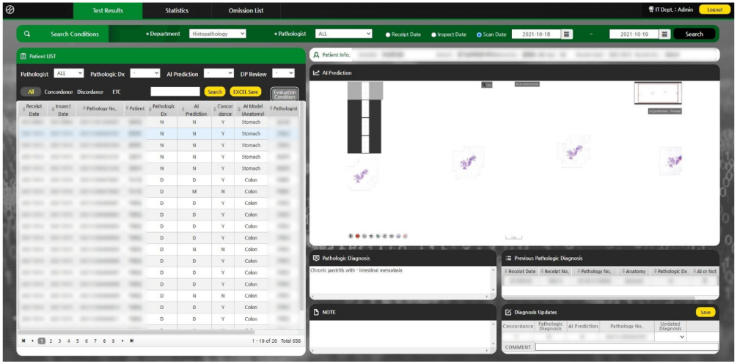


**S1 Fig. Display of an example “Test Results” page in the SeeDP system.** In the SeeDP system, scanned slide data can be searched by receipt date, inspection date, and scan date on the “Test Results” and “Statistics” pages. The “Test Results” page provides the slide information list for each WSI, including the receipt date, inspection date, pathology number (slide name), patient name, classification by pathologic diagnosis, classification by AI prediction, concordance, AI model (anatomy), and pathologist (reader). It also provides text information, including a AI model heatmap thumbnail at single magnification (0.5x), pathologic diagnosis, notes, and previous pathologic diagnoses. **Abbreviations:** AI (artificial intelligence), SeeDP (Seegene Medical Foundation’s AI-assisted Digital Pathology Total Solution), WSI (whole slide image)
