# Supplementary material for: Improving quality control in the routine practice for histopathological interpretation of gastrointestinal endoscopic biopsies using artificial intelligence
Source: PLoS One. 2022 Dec 15;17(12):e0278542. doi: 10.1371/journal.pone.0278542 (PMC9754254; doi:10.1371/journal.pone.0278542)
Supplement: S2 Fig — From the “Statistics” page, the prediction performance and distribution of each AI model (gastric/colorectal) can be checked. Moreover, the pathologist can review the applicable WSIs by selecting the cells within the “AI Distribution” table, which allows the pathologist to selectively review the cases that meet desired conditions from the “Statistics” page, similar to the “Test Results” page. Abbreviations: AI (artificial intelligence), SeeDP (Seegene Medical Foundation’s AI-assisted Digital Pathology Total Solution), WSI (whole slide image). (DOCX) [file pone.0278542.s007.docx]

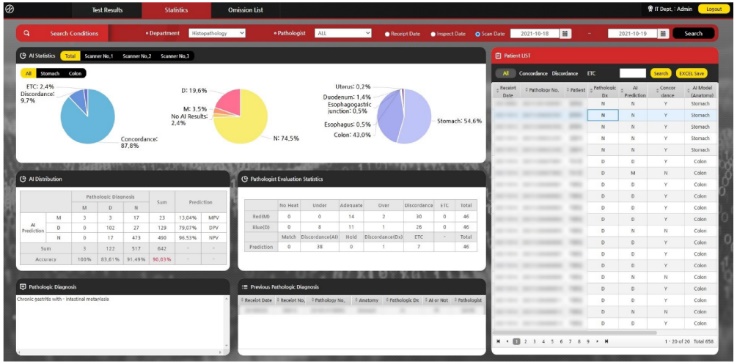


**S2 Fig. An example of the “Statistics” page in the SeeDP system.** From the “Statistics” page, the prediction performance and distribution of each AI model (gastric/colorectal) can be checked. Moreover, the pathologist can review the applicable WSIs by selecting the cells within the “AI Distribution” table, which allows the pathologist to selectively review the cases that meet desired conditions from the “Statistics” page, similar to the “Test Results” page. **Abbreviations:** AI (artificial intelligence), SeeDP (Seegene Medical Foundation’s AI-assisted Digital Pathology Total Solution), WSI (whole slide image)
