# Supplementary material for: Improving quality control in the routine practice for histopathological interpretation of gastrointestinal endoscopic biopsies using artificial intelligence
Source: PLoS One. 2022 Dec 15;17(12):e0278542. doi: 10.1371/journal.pone.0278542 (PMC9754254; doi:10.1371/journal.pone.0278542)
Supplement: S3 Fig — The viewer allows 3DHISTECH WSI files in mrxs file format to be loaded directly; it has a fast image loading speed and excellent scalability because it can be installed in a desktop computer as well as a laptop, mobile phone, or tablet. When the slides list is searched in the SeeDP system and the row of a specific slide is double clicked, the WSI viewer for that slide appears. The image location can be moved by dragging the screen, and the mouse scroll button can be used to zoom in/out, while the mini-map in the upper right corner can be used to identify the location displayed on the screen. Abbreviations: WSI (whole slide image). (DOCX) [file pone.0278542.s008.docx]

**Supporting Information**


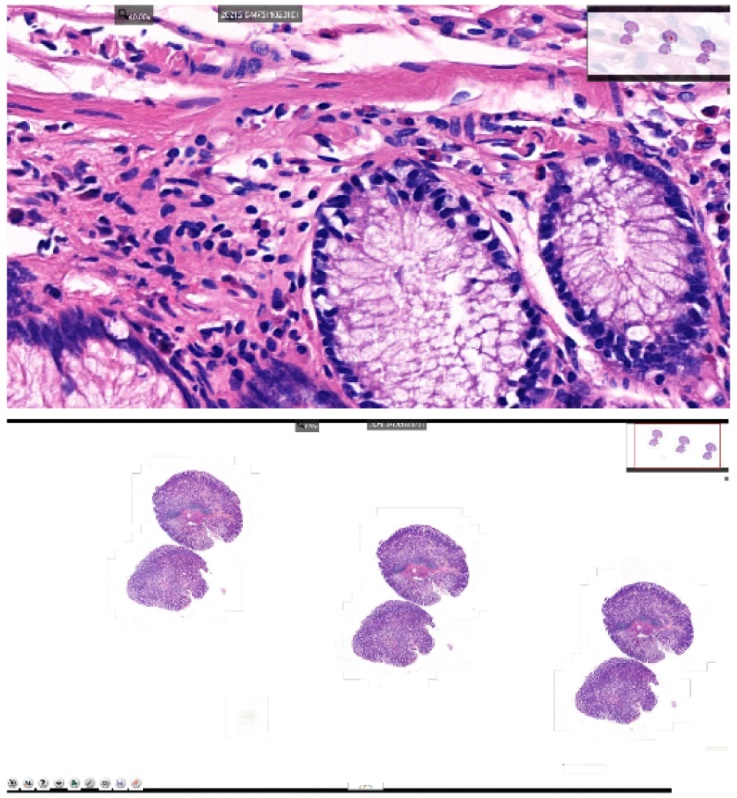


**S3 Fig. Display of the WSI viewer, zoomed in and out.** The viewer allows 3DHISTECH WSI files in mrxs file format to be loaded directly; it has a fast image loading speed and excellent scalability because it can be installed in a desktop computer as well as a laptop, mobile phone, or tablet. When the slides list is searched in the SeeDP system and the row of a specific slide is double clicked, the WSI viewer for that slide appears. The image location can be moved by dragging the screen, and the mouse scroll button can be used to zoom in/out, while the mini-map in the upper right corner can be used to identify the location displayed on the screen. **Abbreviations:** WSI (whole slide image)
