# Supplementary material for: Improving quality control in the routine practice for histopathological interpretation of gastrointestinal endoscopic biopsies using artificial intelligence
Source: PLoS One. 2022 Dec 15;17(12):e0278542. doi: 10.1371/journal.pone.0278542 (PMC9754254; doi:10.1371/journal.pone.0278542)
Supplement: S4 Fig — When there are multiple slides from a single specimen (presence of a recut, serial, or deeper section, or the presence of more than two blocks), the WSI viewer provides a related slide list on the upper left corner to view related WSIs together. Abbreviations: WSI (whole slide image). (DOCX) [file pone.0278542.s009.docx]

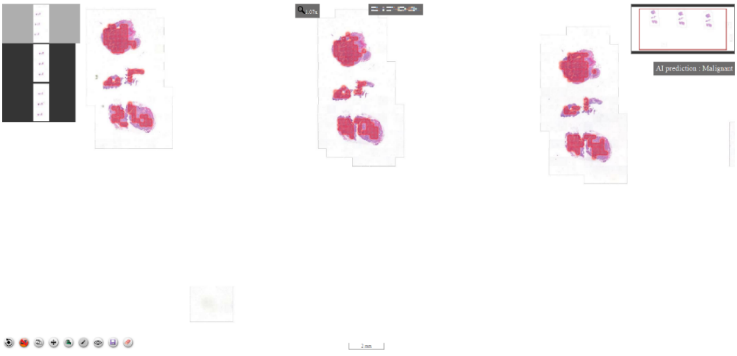


**S4 Fig. Display of serial-section WSIs** When there are multiple slides from a single specimen (presence of a recut, serial, or deeper section, or the presence of more than two blocks), the WSI viewer provides a related slide list on the upper left corner to view related WSIs together. **Abbreviations:** WSI (whole slide image)
