# Supplementary figures and images for: Improving quality control in the routine practice for histopathological interpretation of gastrointestinal endoscopic biopsies using artificial intelligence
Source: PLoS One. 2022 Dec 15;17(12):e0278542. doi: 10.1371/journal.pone.0278542 (PMC9754254; doi:10.1371/journal.pone.0278542)

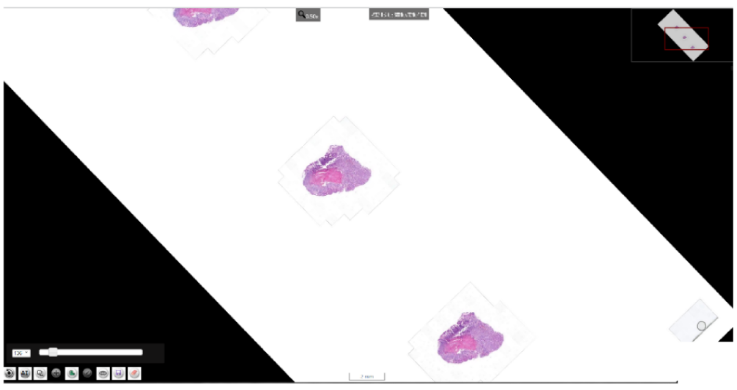


**S5.2 Fig. Rotation of the whole slide image**

Supplement: S5 Fig — S5.1 Fig. Menu buttons. The function buttons in the lower left corner can be used to run various WSI-related functions. From the left, the functions are as follows: perform rotation, visualization of the AI heatmap and prediction, split the screen, position movement, measurement, annotation insertion, annotation lookup, annotation storage, and annotation deletion functions. Abbreviations: AI (artificial intelligence), WSI (whole slide image). S5.2 Fig. Rotation of the whole slide image. S5.3 Fig. Split screen. S5.4 Fig. Annotation. (ZIP) [file pone.0278542.s010.zip › S5.2 Fig.docx]

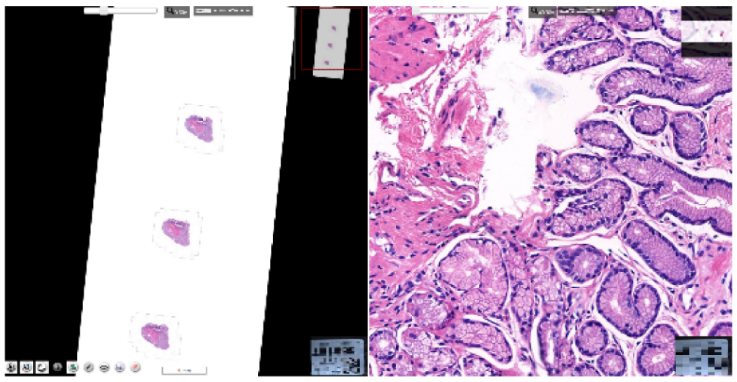


**S5.3 Fig. Split screen**

Supplement: S5 Fig — S5.1 Fig. Menu buttons. The function buttons in the lower left corner can be used to run various WSI-related functions. From the left, the functions are as follows: perform rotation, visualization of the AI heatmap and prediction, split the screen, position movement, measurement, annotation insertion, annotation lookup, annotation storage, and annotation deletion functions. Abbreviations: AI (artificial intelligence), WSI (whole slide image). S5.2 Fig. Rotation of the whole slide image. S5.3 Fig. Split screen. S5.4 Fig. Annotation. (ZIP) [file pone.0278542.s010.zip › S5.3 Fig.docx]

**Supporting Information**


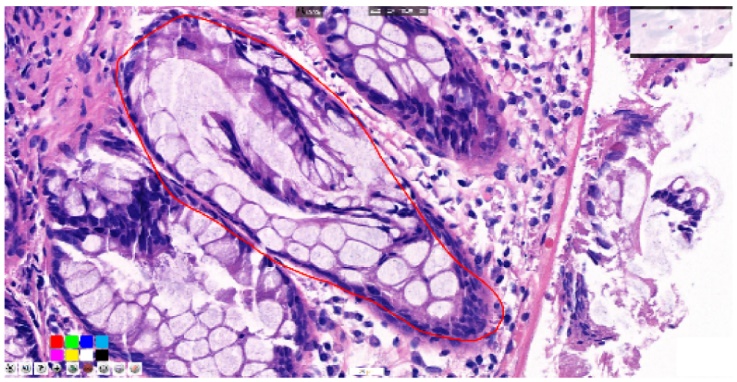


**S5.4 Fig. Annotation**

Supplement: S5 Fig — S5.1 Fig. Menu buttons. The function buttons in the lower left corner can be used to run various WSI-related functions. From the left, the functions are as follows: perform rotation, visualization of the AI heatmap and prediction, split the screen, position movement, measurement, annotation insertion, annotation lookup, annotation storage, and annotation deletion functions. Abbreviations: AI (artificial intelligence), WSI (whole slide image). S5.2 Fig. Rotation of the whole slide image. S5.3 Fig. Split screen. S5.4 Fig. Annotation. (ZIP) [file pone.0278542.s010.zip › S5.4 Fig.docx]
