# Supplementary material for: Improving quality control in the routine practice for histopathological interpretation of gastrointestinal endoscopic biopsies using artificial intelligence
Source: PLoS One. 2022 Dec 15;17(12):e0278542. doi: 10.1371/journal.pone.0278542 (PMC9754254; doi:10.1371/journal.pone.0278542)
Supplement: S6 Fig — We developed a visualization function that would allow slide-level and patch-level information to be viewed on top of a single WSI in the WSI viewer. The final slide-level prediction is marked with a text label on the bottom of the mini-map in the upper right corner of the WSI viewer. Moreover, patch-prediction information is produced in heatmap format, and based on location information for each patch, prediction information is expressed on top of each patch within the WSI. Class M, D, and N patches are expressed as red, blue, and no heat. In particular, heatmap mask-on and -off can be enabled by simply right-clicking while viewing the slides through the WSI viewer. The basic functions of the WSI viewer, such as zoon in/out and rotation, can be used to simultaneously view the WSI and heatmap at all magnifications (0.5x–40x). Accordingly, when a human pathologist reviews WSIs, he or she can intuitively check how AI models inferred the parts of a single WSI while turning the heatmap on and off at all magnifications. Abbreviations: AI (artificial intelligence), WSI (whole slide image), M (Malignant), D (Dysplasia), N (Negative for dysplasia). (DOCX) [file pone.0278542.s011.docx]

**
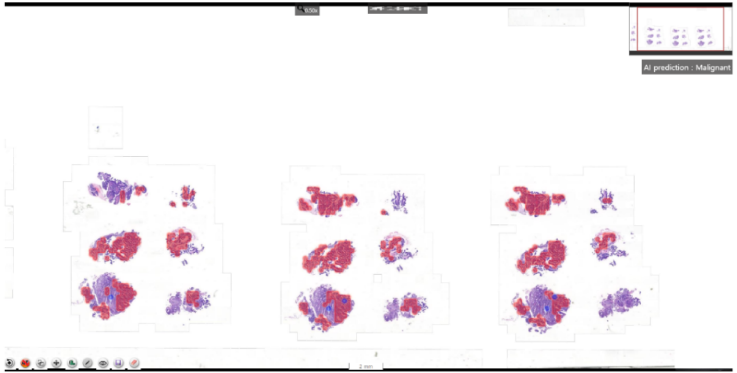
**

S6 Fig. WSI Viewer – Visualization of the heatmap and prediction of the AI model We developed a visualization function that would allow slide-level and patch-level information to be viewed on top of a single WSI in the WSI viewer. The final slide-level prediction is marked with a text label on the bottom of the mini-map in the upper right corner of the WSI viewer. Moreover, patch-prediction information is produced in heatmap format, and based on location information for each patch, prediction information is expressed on top of each patch within the WSI. Class M, D, and N patches are expressed as red, blue, and no heat. In particular, heatmap mask-on and -off can be enabled by simply right-clicking while viewing the slides through the WSI viewer. The basic functions of the WSI viewer, such as zoon in/out and rotation, can be used to simultaneously view the WSI and heatmap at all magnifications (0.5x–40x). Accordingly, when a human pathologist reviews WSIs, he or she can intuitively check how AI models inferred the parts of a single WSI while turning the heatmap on and off at all magnifications. Abbreviations: AI (artificial intelligence), WSI (whole slide image), M (Malignant), D (Dysplasia), N (Negative for dysplasia)
