# Supplementary material for: Improving quality control in the routine practice for histopathological interpretation of gastrointestinal endoscopic biopsies using artificial intelligence
Source: PLoS One. 2022 Dec 15;17(12):e0278542. doi: 10.1371/journal.pone.0278542 (PMC9754254; doi:10.1371/journal.pone.0278542)
Supplement: S7 Fig — This case was classified as class M by pathologic diagnosis and class N by AI prediction, and was only case of severe discordance. It was revealed that this case was classified as class M based on a diagnosis of “TA, HGD”, but it was an ambiguous case with small lesion size and possible interobserver discrepancy in the dysplasia grading. The AI model showed red heat at the patch-level in the corresponding region, but the final prediction was class N. Abbreviations: AI (artificial intelligence), WSI (whole slide image), M (Malignant), N (Negative for dysplasia), TA (tubular adenoma), HGD (high grade dysplasia). (DOCX) [file pone.0278542.s012.docx]

**
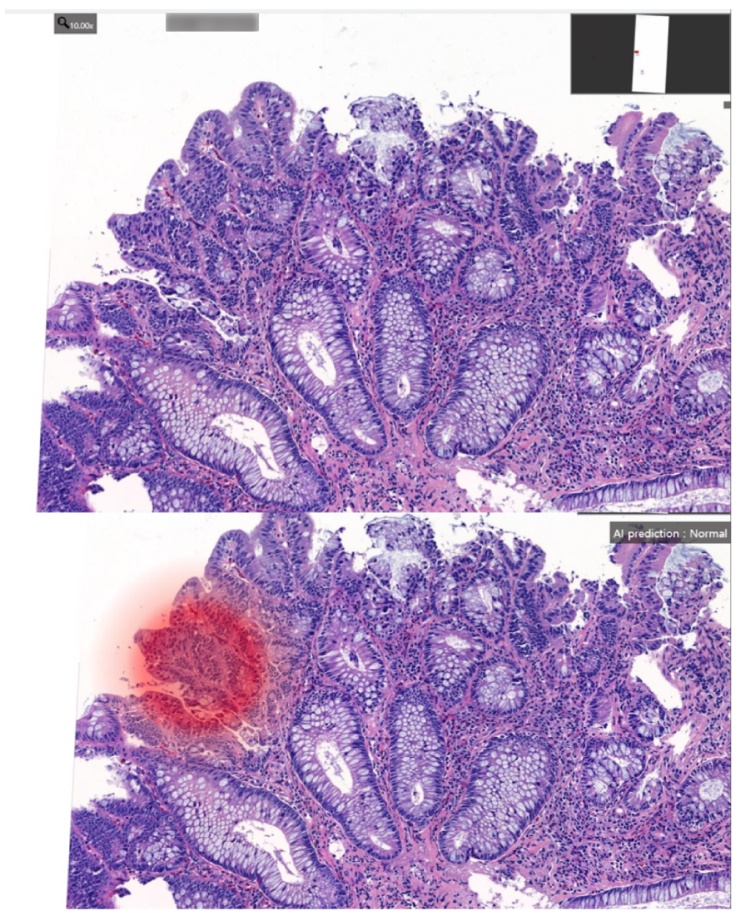
**

**S7 Fig. WSI and heatmap of the only case of severe discordance** This case was classified as class M by pathologic diagnosis and class N by AI prediction, and was only case of severe discordance. It was revealed that this case was classified as class M based on a diagnosis of “TA, HGD”, but it was an ambiguous case with small lesion size and possible interobserver discrepancy in the dysplasia grading. The AI model showed red heat at the patch-level in the corresponding region, but the final prediction was class N. **Abbreviations:** AI (artificial intelligence), WSI (whole slide image), M (Malignant), N (Negative for dysplasia), TA (tubular adenoma), HGD (high grade dysplasia)
