# Supplementary material for: Improving quality control in the routine practice for histopathological interpretation of gastrointestinal endoscopic biopsies using artificial intelligence
Source: PLoS One. 2022 Dec 15;17(12):e0278542. doi: 10.1371/journal.pone.0278542 (PMC9754254; doi:10.1371/journal.pone.0278542)
Supplement: S8 Fig — S8.1 Fig. A representative false positive case (N to M) in the gastric model. Class M prediction with red heat on the ulcer-related change areas. This case was diagnosed as HCG with ulcer. Abbreviations: M (Malignant), N (Negative for dysplasia), TA (tubular adenoma), HCG (H. Pylori-associated chronic gastritis). S8.2 Fig. A representative false positive case (N to D) in the gastric model. Class D prediction with blue heat on the darkly stained and tangentially sectioned foveolar epithelium with knife marks. No heat in the other xanthomatous areas. This case was diagnosed as xanthoma. Abbreviations: D (Dysplasia), N (Negative for dysplasia). S8.3 Fig. A representative false positive case (N to M) in the gastric model. Class M prediction with red heat in erosion-related change areas. This case was diagnosed as xanthelasma with erosion. There was no heat in other xanthomatous areas. Abbreviations: M (Malignant), N (Negative for dysplasia). S8.4 Fig. The only false negative case (D to N) in the gastric model. Class N prediction with focal blue and red heat only in the dysplastic area. This case was diagnosed as TA, LGD. Abbreviations: D (Dysplasia), N (Negative for dysplasia), TA (tubular adenoma), LGD (low grade dysplasia). S8.5 Fig. Representative cases for NET of the stomach and colon. Most of these cases were predicted as class N with no heat in both models. Abbreviations: N (Negative for dysplasia), NET (neuroendocrine tumor). S8.6 Fig. Histopathologic findings of a specific case diagnosed as “favor NET” and predicted as class M in the gastric AI model. While low-magnification findings seemed to show NET, high-magnification findings indicated that this case needed to be differentiated from “oxyntic gland adenoma exhibiting infiltrative growing pattern” and “gastric adenocarcinoma of the fundic-gland type”. Abbreviations: AI (artificial intelligence), M (Malignant), NET (neuroendocrine tumor). S8.7 Fig. Histopathologic findings of a specific case of “s/f MALT lymph [file pone.0278542.s013.zip › S8.2 Fig.docx]

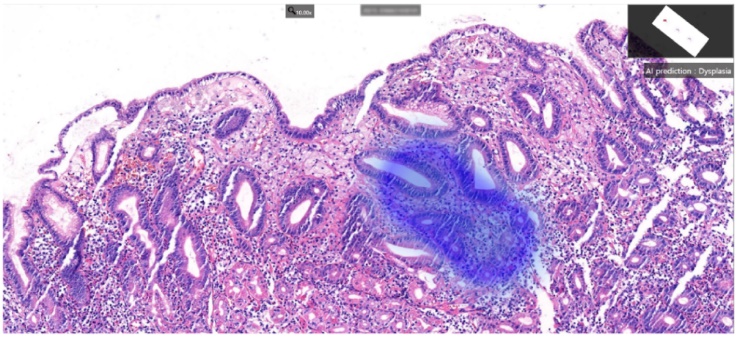


**S8.2 Fig. A representative false positive case (N to D) in the gastric model** Class D prediction with blue heat on the darkly stained and tangentially sectioned foveolar epithelium with knife marks. No heat in the other xanthomatous areas. This case was diagnosed as xanthoma. **Abbreviations:** D (Dysplasia), N (Negative for dysplasia)
